# Mechanical Biomimetic Nanocomposites for Multidimensional Treatment of Arterial Thrombosis

**DOI:** 10.1002/advs.202501134

**Published:** 2025-04-15

**Authors:** Ying Li, Qi Xiang, Yan Zhang, Fuxue Luo, Yunfang Wu, Haitao Ran, Yang Cao

**Affiliations:** ^1^ Chongqing Key Laboratory of Ultrasound Molecular Imaging and Therapy Ultrasound Department of the Second Affiliated Hospital Institute of Ultrasound Imaging State Key Laboratory of Ultrasound in Medicine and Engineering Chongqing Medical University Chongqing 400016 China

**Keywords:** arterial thrombolysis, nitric oxide, photoacoustic imaging, photothermal therapy, Prussian blue nanocomposite, reactive oxygen species

## Abstract

Arterial thrombosis is a severe cardiovascular condition associated with high mortality and disability rates. Traditional pharmacological thrombolytic therapies, however, are limited by a narrow therapeutic window, short drug half‐lives, restricted therapeutic effectiveness, and the risk of hemorrhagic complications. Moreover, the unique biological microenvironment of arterial thrombosis is characterized by high shear stress and an inflammatory milieu rich in reactive oxygen species (ROS), resulting in a relatively high recurrence rate. To address these challenges, a mechanical biomimetic nanocomposite MPB‐NO‐UK@PM is developed by doping sodium nitroprusside, a nitric oxide (NO) donor, into mesoporous Prussian blue (MPB) nanoparticles to form MPB‐NO, which is then loaded with urokinase (UK) and coated with platelet membranes (PM). The nanocomposite exhibits excellent thrombus‐targeting ability and enhances thrombolytic efficacy through synergistic photothermal and pharmacological, while simultaneously reducing the risk of bleeding. The released NO induces antiplatelet aggregation effects, while Prussian blue effectively scavenges ROS and inflammatory cytokines, thereby preventing thrombus recurrence. Additionally, the nanocomposite possesses superior photoacoustic imaging capabilities, enabling visualization of thrombus diagnosis and treatment. This therapeutic approach represents a novel pathway for the multidimensional and efficient management of arterial thrombosis.

## Graphical abstract

In this study, a mechanical biomimetic nanocomposite is developed, guided by photoacoustic imaging, to achieve highly efficient and safe thrombolysis through the synergistic effects of photothermal therapy and pharmacological intervention. Most notably, the nanoparticle improves the microenvironment of arterial thrombi, marked by high shear stress and ROS, effectively reducing thrombus recurrence risk.

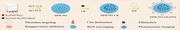

## 1 Introduction

Arterial thrombosis, primarily induced by the rupture or erosion of atherosclerotic plaques, is a serious cardiovascular condition that presents significant challenges to global public health due to its high rates of mortality and disability.^[^ ^1^ ^]^ When atherosclerotic plaques rupture or erode, platelets rapidly aggregate and interact with coagulation factors such as fibrin, leading to the formation of thrombi that obstruct the arterial lumen and result in severe consequences, including myocardial infarction and stroke.^[^ ^2^, ^3^ ^]^ The most commonly used pharmacological thrombolytic therapy in clinical practice involves the administration of tissue plasminogen activator (tPA); however, it is limited by a narrow therapeutic window, a short drug half‐life, limited therapeutic efficacy, and the risk of hemorrhagic complications.^[^ ^4^ ^]^ Following thrombolytic therapy, residual thrombus fragments and activated platelets within the blood vessels may reaggregate, leading to vascular reocclusion, which occurs with an incidence rate of ≈25–30%.^[^ ^5^ ^]^ While antiplatelet drugs can inhibit platelet aggregation and thereby reduce the risk of recurrence, they also increase the risk of bleeding.^[^ ^6^ ^]^ Consequently, there is an urgent need to develop a comprehensive therapeutic strategy for arterial thrombosis that is effective in thrombolysis, prevents recurrence, and carries a low risk of bleeding.

With the emergence of smart responsive nanomaterials, targeted nanodrug delivery systems have undergone significant advancements in preclinical studies for thrombus treatment.^[^ ^7^, ^8^ ^]^ These systems typically employ nanomaterials as carriers to encapsulate commonly used thrombolytic drugs in clinical practice, such as urokinase (UK) or recombinant tissue plasminogen activator (rt‐PA), while enhancing targeting through physical methods and biological modifications. This approach aims to improve drug stability within the body, prolong circulation time, increase drug accumulation at the thrombus site, and, consequently, enhance therapeutic efficacy.^[^ ^9^, ^10^ ^]^ Photothermal therapy involves converting optical energy into hyperthermic energy via the Landau damping effect under near‐infrared light irradiation, which physically eradicates fibrin clots.^[^ ^11^, ^12^ ^]^ Furthermore, the local temperature increase induced by the photothermal effect accelerates the motion of molecules within and around the nanocarriers or triggers alterations in the nanomaterial structure or chemical bonds, facilitating the release of drug molecules from the carriers.^[^ ^13^, ^14^ ^]^ The integration of photothermal therapy with targeted nanodrug delivery systems has emerged as an efficient and non‐invasive thrombolytic strategy, becoming a prominent research focus in thrombus treatment.^[^ ^15^, ^16^ ^]^ However, despite the improvement in embolic symptoms, these therapeutic strategies often neglect the biomicroenvironmental characteristics of arterial thrombi, including the high‐shear stress physical environment and the inflammatory milieu rich in reactive oxygen species (ROS), which may contribute to thrombus recurrence.

Arterial thrombi are characterized by a physical environment that exhibits high shear stress.^[^ ^17^ ^]^ The formation of an arterial thrombus can lead to significant luminal narrowing, accompanied by heightened vascular constriction, resulting in a substantial increase in local blood flow shear stress, which may increase by one or two orders of magnitude.^[^ ^18^, ^19^ ^]^ The shear stress generated by blood flow exerts a force on the surface of vascular endothelial cells, inducing changes in cell membrane tension. Piezo1, a mechanically sensitive cation‐selective channel, responds to mechanical stimuli applied to the cell membrane.^[^ ^20^, ^21^ ^]^ Activation of the Piezo1 channel triggers a rapid influx of calcium ions (Ca^2^⁺) into the cell in response to shear stress; however, this response can vary significantly depending on the intensity of the applied shear stress. At lower levels of shear stress, channel activity may gradually increase, whereas, at higher levels, the response may become saturated or even lead to channel inactivation, resulting in a reduced Ca^2^⁺ influx despite ongoing mechanical stimulation.^[^ ^22^, ^23^ ^]^ Endothelial nitric oxide synthase (eNOS) is a key enzyme responsible for the production of nitric oxide (NO), and its activation mechanism is positively regulated by intracellular Ca^2^⁺ levels influenced by Piezo1 channel activity.^[^ ^24^ ^]^ Therefore, we can infer that the sharply elevated shear stress at the site of arterial thrombi leads to decreased Piezo1 channel activity, which in turn causes reduced Ca^2^⁺ influx in endothelial cells, limiting eNOS activation and decreasing NO production. As an important biological messenger molecule, NO plays a crucial role in regulating thrombus formation by inhibiting platelet adhesion and aggregation, thereby reducing the risk of thrombus formation.^[^ ^25^, ^26^, ^27^ ^]^ Furthermore, nanotechnology‐based NO delivery systems facilitate targeted delivery and on‐demand release of NO, significantly enhancing its antithrombotic effect.^[^ ^28^, ^29^, ^30^ ^]^

An inflammatory environment characterized by an abundance of ROS also facilitates the malignant progression of arterial thrombosis.^[^ ^31^, ^32^ ^]^ The activation of platelets and damage to endothelial cells result in the release of various active substances and inflammatory cytokines, which promote platelet adhesion and aggregation while simultaneously stimulating the production of significant amounts of ROS.^[^ ^33^, ^34^ ^]^ In turn, ROS enhance platelet activation and recruitment by influencing multiple cellular functions and signaling pathways, and they also stimulate the overexpression of inflammatory cytokines in vascular endothelial cells.^[^ ^35^ ^]^ Shear stress, particularly the elevated shear stress observed in arterial thrombi, accelerates ROS production, thereby intensifying the signaling cascade that leads to platelet activation and aggregation, which further exacerbates thrombus formation.^[^ ^33^, ^36^ ^]^

**Scheme 1:**
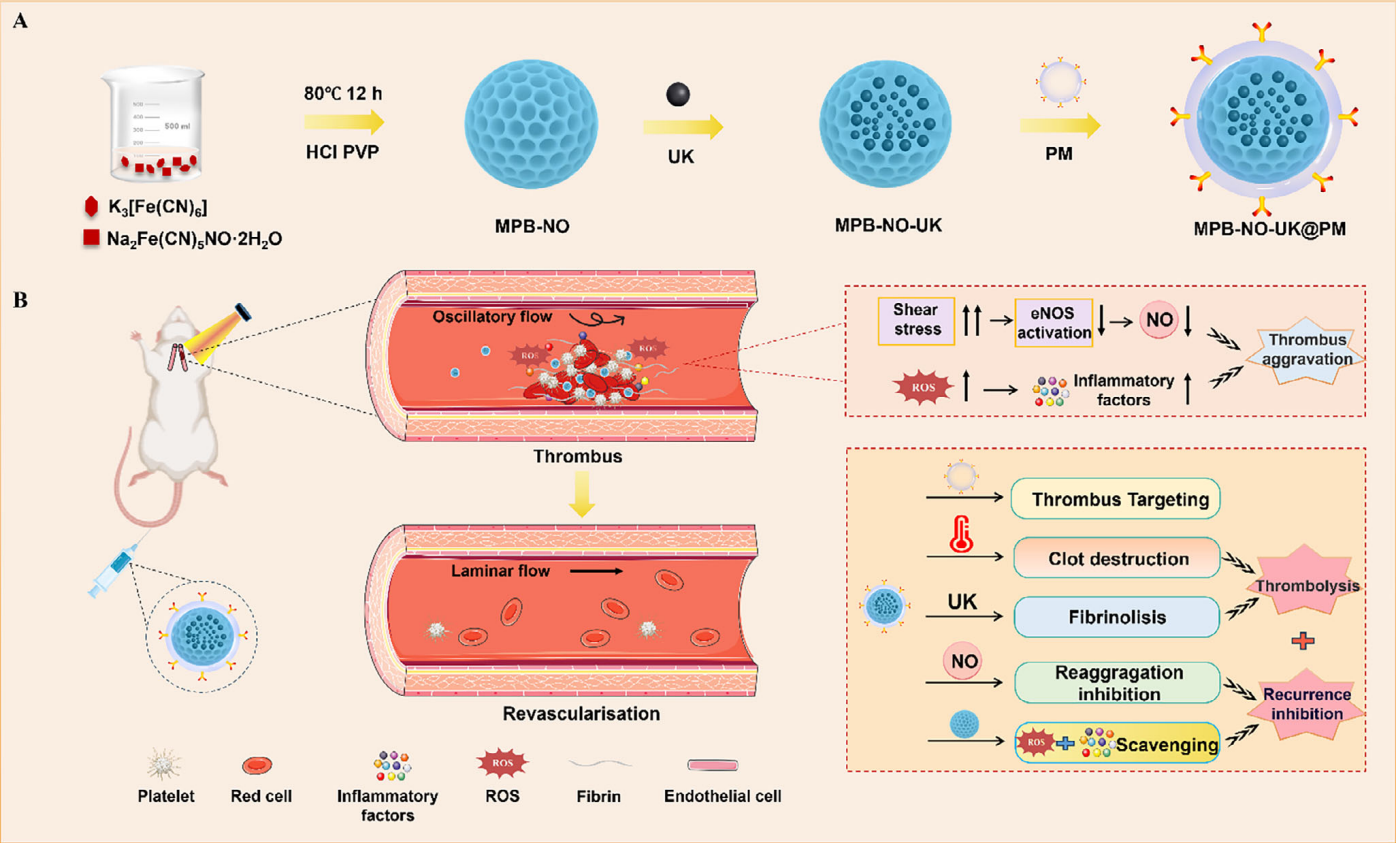
Schematic diagram of the multidimensional treatment of arterial thrombosis facilitated by MPB‐NO‐UK@PM nanocomposites.

Considering the biomicroenvironmental characteristics of arterial thrombi discussed above, we have designed a thrombus‐targeted nanocomposite aimed at achieving photothermal‐synergistic drug‐induced thrombolysis. This approach also seeks to replenish the NO deficiency resulting from decreased Piezo1 channel activation and to eliminate excess ROS in the inflammatory environment, thereby preventing thrombus recurrence. Prussian blue, recognized as an antidote for heavy metal poisoning, has been approved by the U.S. Food and Drug Administration (FDA) and demonstrates excellent biocompatibility and safety in clinical applications.^[^ ^37^ ^]^ In recent years, mesoporous Prussian blue (MPB) nanoparticles have garnered considerable attention due to their unique properties and potential applications in various fields, including catalysis, drug delivery, and photothermal therapy. Sodium nitroprusside (SNP) is a widely utilized medication for the management of hypertensive emergencies and acute left heart failure. It is metabolized by vascular smooth muscle to produce NO and exhibits a notable chemical structural similarity to potassium ferricyanide, which is the primary synthetic precursor for the preparation of MPB.^[^ ^38^, ^39^ ^]^ As illustrated in **Scheme** **1**, potassium ferricyanide (K_3_[Fe(CN)_6_]) and SNP (Na_2_[Fe(CN)_5_NO]·2H_2_O) are co‐stirred in an acidic and heated environment. The nitroso group in SNP connects to the Fe^3+^ in potassium ferricyanide through a coordination bond, forming MPB‐NO nanoparticles with a mesoporous structure. Subsequently, the thrombolytic drug UK is loaded into the MPB‐NO nanoparticles, and the surface is coated with platelet membranes (PM), producing the multifunctional nanocomposite MPB‐NO‐UK@PM for the treatment of arterial thrombi. Following intravenous infusion, this nanocomposite targets activated platelets and damaged endothelial cells due to the platelet membrane coating. Under the irradiation of an 808 nm laser, localized heating triggers the release of UK and NO, achieving dual thrombolytic effects through photothermal therapy and drug delivery. Concurrently, it replenishes NO in the microenvironment and eliminates ROS to prevent thrombus recurrence. Furthermore, this nanocomposite exhibits excellent photoacoustic imaging capabilities, enhancing the accuracy of thrombus detection and facilitating the visualization of thrombus diagnosis and treatment. This treatment strategy paves a new path toward comprehensive and efficient management of arterial thrombi.

## 2 Results and Discussion

### 2.1 Characterization of MPB‐NO‐UK@PM

**Figure 1:**
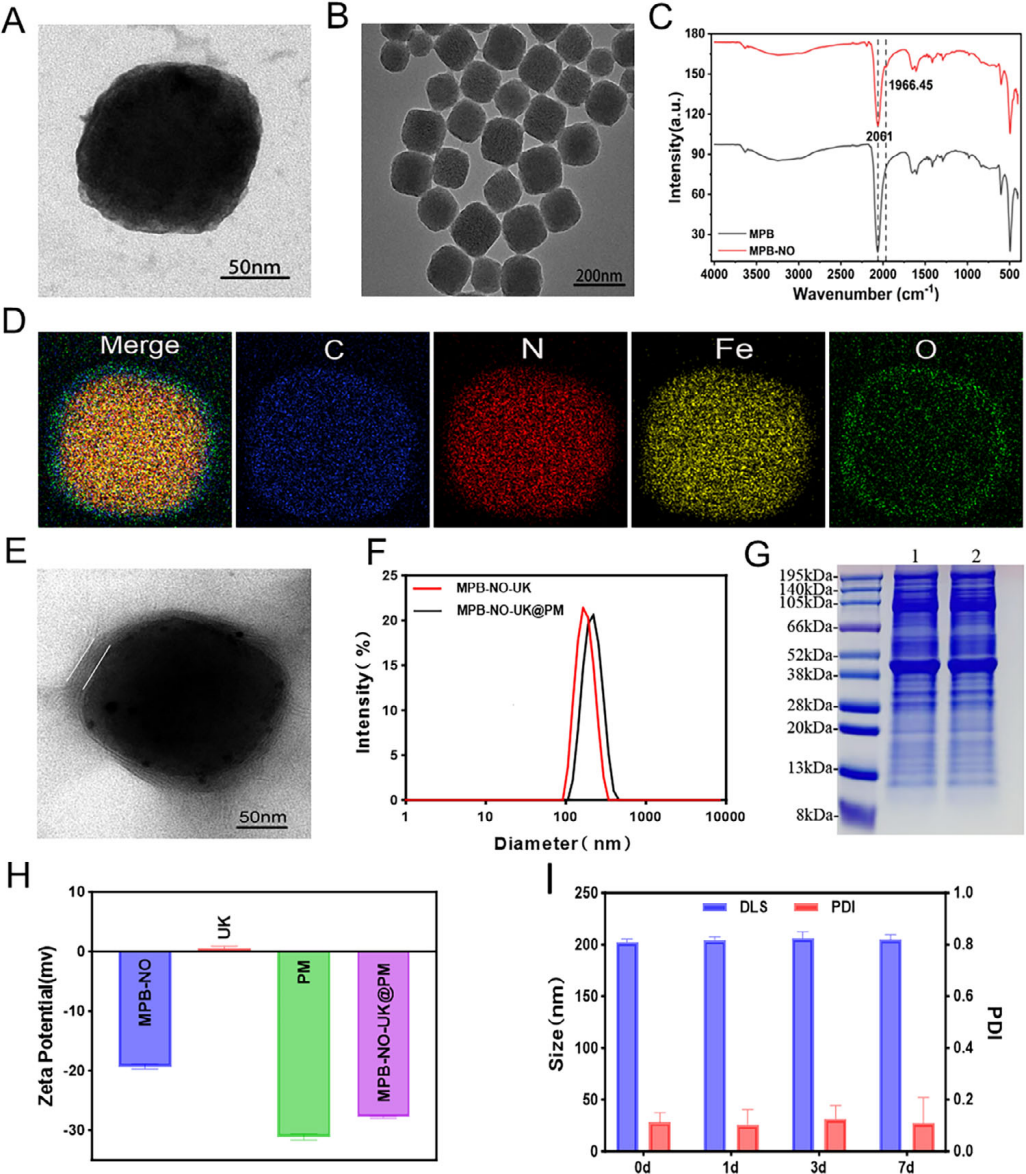
Characterization of nanocomposites. A,B) TEM and HRTEM images of MPB‐NO. C) FTIR spectrums of MPB‐NO and MPB. D) Elemental mapping of MPB‐NO. E) TEM images of MPB‐NO‐UK@PM. F) Size distribution of MPB‐NO‐UK and MPB‐NO‐UK@PM. G) SDS‐PAGE analysis of PM and MPB‐NO‐UK@PM. Note: 1 denotes PM and 2 stands for MPB‐NO‐UK@PM. H) Zeta potential of MPB‐NO, UK, PM, MPB‐NO‐UK@PM (*n* = 3). I) Size distribution and PDI of MPB‐NO‐UK@PM over one week (*n* = 3).

Based on methods reported in previous studies,^[^ ^38^, ^39^ ^]^ SNP was embedded into the MPB framework under acidic conditions to obtain MPB‐NO. UK was successfully loaded into the mesoporous structure of MPB‐NO. Following the coating of the nanoparticle surface with platelet membranes, the composite MPB‐NO‐UK@PM was obtained. Transmission electron microscopy (TEM) and high‐resolution transmission electron microscopy (HRTEM) images (**Figure** **1**A,B) revealed that MPB‐NO consists of uniform square‐shaped particles with a diameter of ≈150 nm. Fourier transform infrared spectroscopy (FTIR) demonstrated an additional N═O vibration peak at 1966 cm⁻^1^ in the MPB‐NO spectrum, compared to the MPB spectrum (Figure 1C), indicating the successful incorporation of SNP into the MPB framework to form MPB‐NO. To further confirm the successful preparation of MPB‐NO, the presence of nitrosyl groups in MPB‐NO was demonstrated through X‐ray photoelectron spectroscopy (XPS) and element mapping analysis. The XPS spectra (Figure S1, Supporting Information) indicate that the binding energies of the Fe (2P) characteristic peaks for both MPB and MPB‐NO are observed at 708 eV (2P_2/3_) and 721 eV (2P_1/3_), respectively, while the C (1s) characteristic peak shows a binding energy of 284 eV. For N (1s), the peak at 396 eV corresponds to the cyano group (C≡N) present in both MPB and MPB‐NO, whereas the peak at 399 eV specifically indicates the nitroso (N═O) functional group in MPB‐NO. Furthermore, the elemental mapping of MPB‐NO (Figure 1D) confirms the uniform distribution of oxygen within the nanostructure. Additionally, X‐ray diffraction (XRD) results (Figure S2, Supporting Information) demonstrate that MPB‐NO exhibits the same diffraction peaks as MPB, suggesting that the incorporation of SNPs does not alter the crystal structure of MPB. The N_2_ adsorption–desorption isotherms (Figure S3, Supporting Information) reveal that MPB‐NO nanoparticles possess a type IV mesoporous structure with a specific surface area of ≈125.1 m^2^ g⁻^1^, indicating their excellent drug‐loading capacity. Subsequently, the UK was encapsulated into MPB‐NO to produce MPB‐NO‐UK. Based on the UK standard curve measured using the UK Elisa kit (Figure S4, Supporting Information), the encapsulation efficiency of the UK in MPB‐NO was calculated to be 60.83 ± 2.08%, with a drug loading efficiency of 10.84 ± 0.33%. The core–shell structure of MPB‐NO‐UK@PM was observed using transmission electron microscopy (Figure 1E), confirming the successful coating of the platelet membrane onto the surface of the nanoparticles. Additionally, dynamic light scattering (DLS) results indicated that the average diameter of MPB‐NO‐UK was 182.2 ± 4.7 nm, which increased to 202.6 ± 2.9 nm following the coating with PM (Figure 1F). Furthermore, sodium dodecyl sulfate‐polyacrylamide gel electrophoresis (SDS‐PAGE) demonstrated that the protein electrophoresis bands of the platelet membrane on MPB‐NO‐UK@PM were largely consistent with those of the source platelet membrane (Figure 1G), suggesting that the platelet membrane coating on the nanoparticles retained its integrity and could effectively exert its original biological functions. Based on DLS measurements, the zeta potentials of MPB‐NO, UK, PM, and MPB‐NO‐UK@PM were −19.3 ± 0.4, 0.53 ± 0.37, −31.1 ± 0.529, and −27.8 ± 0.2, respectively (Figure 1H). These results indicated that the potentials of MPB‐NO and UK were opposite, facilitating increased drug loading efficiency of UK through electrostatic adsorption. These findings further corroborated the successful coating of the platelet membrane. Additionally, the particle size and dispersion of MPB‐NO‐UK@PM nanoparticles remained stable over a one‐week period (Figure 1I).

### 2.2 Photothermal Effect and Photoacoustic Imaging of MPB‐NO‐UK@PM In Vitro

**Figure 2:**
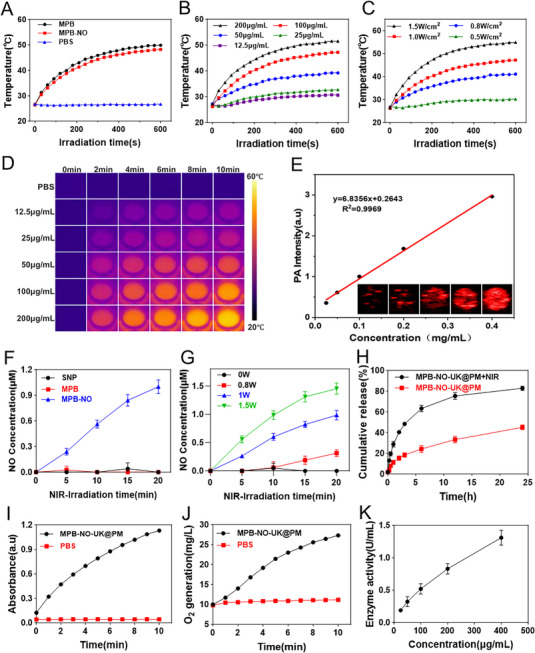
Some properties of MPB‐NO‐UK@PM in vitro. A) Temperature variation curves of MPB, MPB‐NO, PBS. B) Temperature variation curves of MPB‐NO‐UK@PM at various concentrations. C) Temperature elevation curves of with various laser powers. D) Thermal images of MPB‐NO‐UK@PM at different concentrations. E) Quantitative analysis of the signal‐concentration relationship in vitro, with inset corresponding PA images of MPB‐NO‐UK@PM at different concentrations. F) NO release profiles of SNP, MPB, and MPB‐NO exposing to an 808 nm laser at a power density of 1 W cm⁻^2^ (*n* = 3). G) NO release profiles of MPB‐NO‐UK@PM exposing to an 808 nm laser at various power densities (*n* = 3). H) UK release from MPB‐NO‐UK@PM with or without laser irradiation (*n* = 3). I) Absorbance at 650 nm following the addition of TMB substrate solution. J) O_2_ concentration from dissolved oxygen meter. K) SOD activity of MPB‐NO‐UK@PM at various concentrations.

To investigate the photothermal properties of MPB‐NO, we exposed it to an 808 nm near‐infrared (NIR) laser with a power density of 1 W cm⁻^2^ for 10 min. Control groups included an MPB solution and a Phosphate Buffered Saline (PBS) buffer. Simultaneously, we employed an infrared thermal imaging camera to monitor the temperature changes of the solutions. The results demonstrated that after 10 min of irradiation, the temperatures of MPB‐NO and MPB increased by 21.8 and 23.3 °C, respectively, while the temperature of PBS exhibited no significant change (**Figure** **2**A). These findings indicate that MPB‐NO possesses favorable photothermal effects, although they are slightly weaker than those of MPB, potentially due to the higher absorbance of MPB at 808 nm compared to MPB‐NO. Subsequently, MPB‐NO‐UK@PM at varying concentrations was exposed to the 808 nm laser, and the infrared thermal imaging camera revealed that the temperature increased with the concentration of MPB‐NO‐UK@PM (Figure 2B,D). Furthermore, when MPB‐NO‐UK@PM at a specific concentration (100 µg mL^−1^) was exposed to 808 nm NIR lasers of varying power densities, the temperature rose with increasing power (Figure 2C). Additionally, MPB‐NO‐UK@PM exhibited excellent photothermal stability after four ON/OFF heating cycles (Figure S5, Supporting Information).

The photoacoustic imaging system demonstrated that MPB‐NO‐UK@PM solution exhibited the optimal photoacoustic excitation effect at a wavelength of 710 nm (Figure S6, Supporting Information), with the intensity of the photoacoustic signal increasing linearly in relation to the concentration of MPB‐NO‐UK@PM (Figure 2E).

### 2.3 NIR‐Responsive Release of NO and UK In Vitro

To investigate whether NIR laser can effectively trigger the release of NO from MPB‐NO, this study analyzed the NIR‐responsive NO release of MPB‐NO in an in vitro environment and monitored the dynamics of NO release using the Griess assay. The results presented in Figure 2F indicate that, in contrast to SNP and MPB which exhibit no significant response to NIR laser irradiation, MPB‐NO possesses the ability to release NO upon exposure to NIR laser. This phenomenon indicates that the photothermal effect generated by MPB‐NO under laser irradiation results in an increase in temperature, which disrupts the coordination bond between the nitrosyl group and Fe^3+^, thereby facilitating the release of NO. In contrast, SNP lacks a photothermal conversion effect and therefore does not exhibit significant NO release behavior, even under laser irradiation. Furthermore, we explored the NO release characteristics of the MPB‐NO‐UK@PM complex under NIR laser irradiation at varying power densities. The findings indicate that the amount of NO released from MPB‐NO increases with both the elevation of laser power density and the extension of irradiation time (Figure 2G). This confirms that precise control over the intensity and duration of NIR laser exposure can effectively regulate the amount of NO released.

Next, we investigated the drug release characteristics of MPB‐NO‐UK@PM upon NIR stimulation and quantified the released UK using a UK ELISA kit. The experimental results demonstrated that after 24 h of irradiation with an 808 nm laser, MPB‐NO‐UK@PM released up to 82.7% of the UK. In contrast, the release amount was only 45% under conditions without laser irradiation (Figure 2H). These data provide strong evidence that NIR irradiation can effectively enhance the release rate of UK from MPB‐NO‐UK@PM, thereby opening up the possibility of achieving more significant fibrinolytic effects in vivo.

### 2.4 ROS Scavenging Properties of MPB‐NO‐UK@PM In Vitro

The TMB colorimetric reaction demonstrated that the absorbance of the MPB‐NO‐UK@PM samples was significantly enhanced compared to the PBS control group, and the peroxidase‐like (POD‐like) activity increased gradually over time (Figure 2I). Measurements obtained using a dissolved oxygen meter revealed a substantial increase in O_2_ production within the H_2_O_2_ + MPB‐NO‐UK@PM reaction system, whereas O_2_ production was not observed in the pure H_2_O_2_ solution. Additionally, the catalase‐like (CAT‐like) activity exhibited a gradual upward trend over time (Figure 2J). Results from the WST‐8 assay indicated that superoxide dismutase‐like (SOD‐like) activity showed an increasing trend with the elevation of MPB‐NO‐UK@PM concentration (Figure 2K).

### 2.5 Effect of Shear Force on Piezo1 Activation

**Figure 3:**
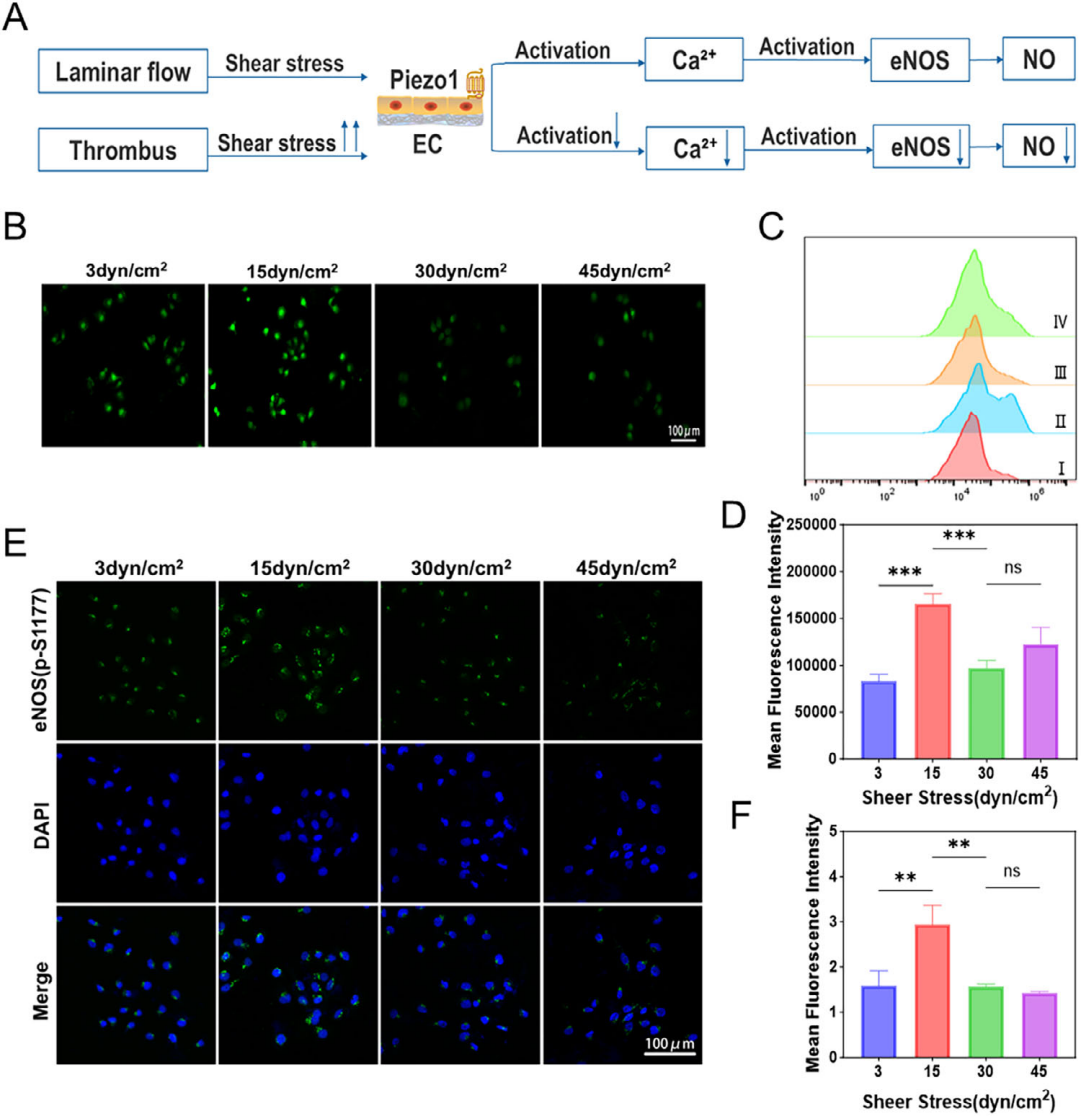
Effects of different shear stresses on Piezo1 channel activation and Ca^2^⁺ influx. A) Schematic diagram illustrating the relationship among shear stress, Piezo1 channel activation, eNOS activation, and NO production. B) Intracellular Ca^2^⁺ concentration following stimulation with different shear stresses observed by Fluo‐4 AM assisted fluorescence microscope. C) Flow cytometry of intracellular Ca^2^⁺ concentration following stimulation with various shear stresses. D) Fluorescence intensity analysis of flow cytometry (*n* = 3). E) CLSM images of phospho‐eNOS(Ser1177) level in HUVECs. F) Mean fluorescence signal of the corresponding phospho‐eNOS (Ser1177) level (*n* = 3). **P < 0.01, ***P < 0.001, n.s.: no significance.

We predict that, as illustrated in **Figure** **3**A, under normal laminar shear stress, Piezo1 channels are activated, resulting in increased Ca^2^⁺ influx in endothelial cells. This influx subsequently activates eNOS, promoting the production of NO, which exerts vasodilatory effects and helps prevent thrombosis. However, the drastically elevated shear stress observed at arterial thrombosis sites leads to a decrease in Piezo1 channel activity, thereby reducing Ca^2^⁺ influx in endothelial cells and limiting eNOS activation, which results in decreased NO production. To verify this theory, we initially employed an inverted fluorescence microscope to investigate the effects of varying magnitudes of shear stress on Ca^2^⁺ levels in human umbilical vein endothelial cells (HUVECs). As shown in Figure 3B, the strongest fluorescent signal was recorded at a shear stress of 15 dyn cm⁻^2^, while further increases in shear stress to 30 and 45 dyn cm⁻^2^ resulted in a significant decline in intracellular fluorescence intensity. To quantitatively assess intracellular Ca^2^⁺ levels, we employed flow cytometry. The results, presented in Figure 3C,D, demonstrate that as shear stress increased from 3 to 15 dyn cm⁻^2^, intracellular Ca^2^⁺ levels also rose. However, when shear stress was elevated to 30 and 45 dyn cm⁻^2^, intracellular Ca^2^⁺ levels began to decline. Subsequently, we assessed the impact of varying magnitudes of shear stress on eNOS activation (phosphorylation of eNOS) through immunofluorescence experiments. As shown in Figure 3E,F, the level of phospho‐eNOS (Ser1177) increased with shear stress from 3 to 15 dyn cm⁻^2^. However, with further increases in shear stress, the level of phospho‐eNOS (Ser1177) decreased. This trend is consistent with the previous experimental results. These findings confirm that a substantial increase in shear stress leads to decreased Piezo1 channel activity, resulting in reduced Ca^2^⁺ influx in endothelial cells, which subsequently decreases eNOS activation and ultimately reduces NO production.

### 2.6 Intracellular Uptake and Adhesion Properties

**Figure 4:**
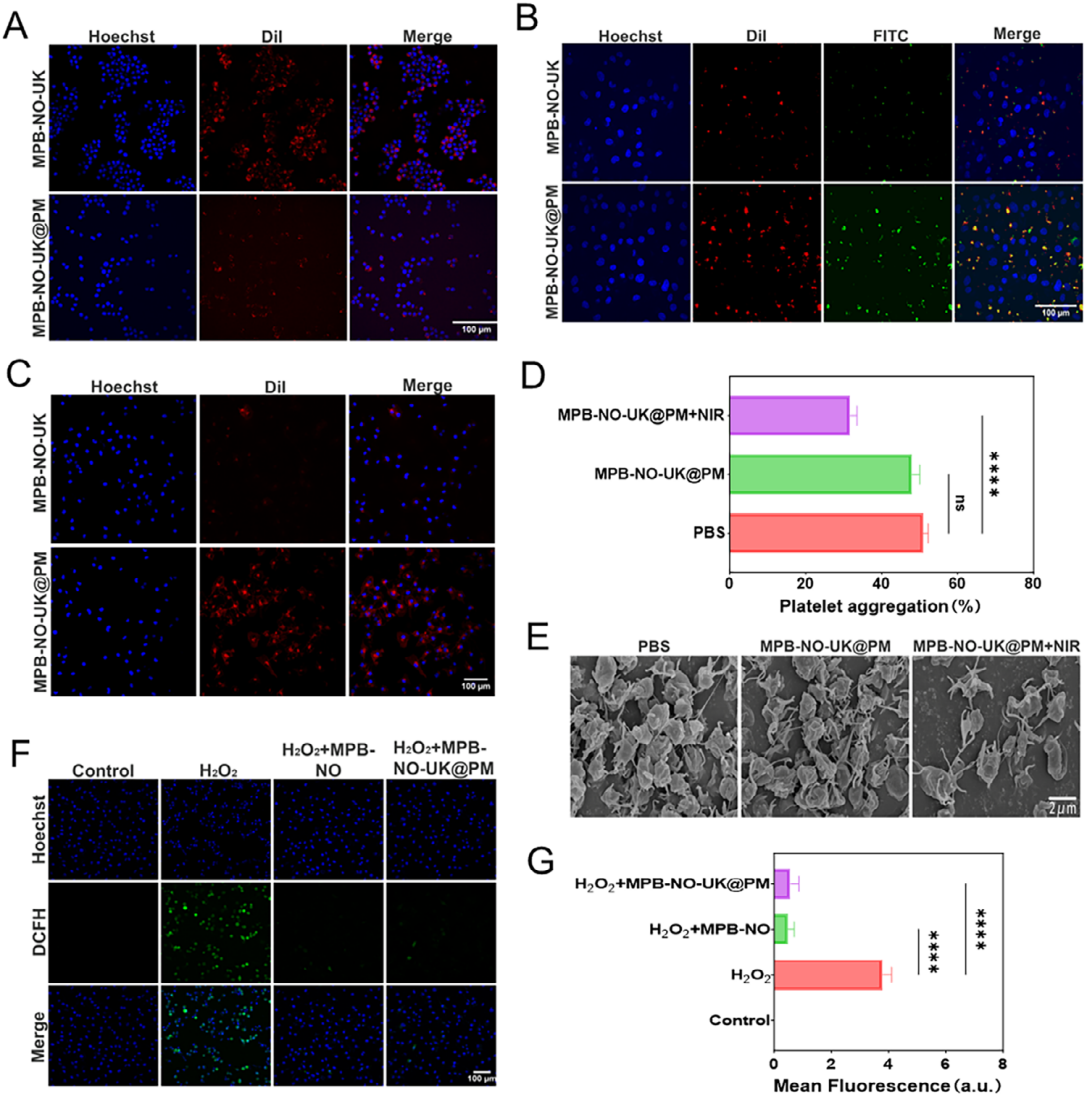
Intracellular uptake and adhesion properties, anti‐platelet aggregation capacity, and H₂O₂ scavenging capacity. A) CLSM images of phagocytosis escape of MPB‐NO‐UK@PM in RAW 264.7 cells. B) CLSM images of MPB‐NO‐UK@PM targeted to activated platelets. C) CLSM images of MPB‐NO‐UK@PM targeted to activated HUVECs. D) Anti‐platelet aggregation effect of NO generated under NIR irradiation (*n* = 3). E) TEM images of the degree of platelet aggregation. F) ROS scavenging in HUVECs following various treatments. G) Mean fluorescence signal of the corresponding ROS level (*n* = 3). ****P < 0.0001, n.s.: no significance.

The CD47 protein present on the platelet membrane is a transmembrane protein that plays a crucial role in immune evasion, diminishing the immune response of macrophages and endowing platelet membrane‐coated nanoparticles with immune‐evasive capabilities.^[^ ^40^ ^]^ We co‐cultured uncoated MPB‐NO‐UK nanoparticles and platelet membrane‐coated MPB‐NO‐UK@PM nanoparticles with RAW264.7 cells for 4 h. Confocal laser scanning microscopy (CLSM) results revealed that, compared to the MPB‐NO‐UK‐treated group, macrophages exhibited strong red fluorescence signals, whereas macrophages in the MPB‐NO‐UK@PM‐treated group displayed only weak red fluorescence (**Figure** **4**A). This comparison demonstrates that MPB‐NO‐UK@PM nanoparticles, after being coated with platelet membranes, can effectively resist uptake by macrophages at the cellular level, thereby promoting prolonged circulation time in vivo.

Nanoparticles coated with platelet membranes can actively bind to specific receptors on the surfaces of activated platelets and activated endothelial cells through membrane proteins such as CD62p, GPVI, and GPlb *α* present on the platelet membrane.^[^ ^41^ ^]^ This interaction significantly enhances targeted delivery efficiency. To investigate the adhesion ability of nanocomposites to activated platelets and activated endothelial cells, CLSM was employed to observe the specific interactions. Initially, adherent HUVECs were incubated with DiI‐labeled activated platelets, while MPB‐NO‐UK or MPB‐NO‐UK@PM were labeled with FITC and subsequently co‐incubated with the cells. As illustrated in Figure 4B, the fluorescent labels of MPB‐NO‐UK@PM largely overlapped with those of activated platelets, whereas the overlap between MPB‐NO‐UK and activated platelets was less pronounced. Furthermore, DiI‐labeled MPB‐NO‐UK and MPB‐NO‐UK@PM were co‐incubated separately with activated or non‐activated HUVECs. The results indicated that the accumulation of MPB‐NO‐UK@PM on activated HUVECs was significantly greater than that of MPB‐NO‐UK (Figure 4C), while no significant difference in fluorescent intensity was observed between the two on non‐activated HUVECs (Figure S7, Supporting Information).

### 2.7 Anti‐Platelet Aggregation Capacity

To investigate the anti‐platelet aggregation effect of MPB‐NO‐UK@PM mediated by NO release, we employed a turbidimetric assay using a microplate reader to measure the platelet aggregation rate. The results (Figure 4D) indicated that the platelet aggregation rates were 50.9 ± 1.3% for the MPB‐NO‐UK@PM group and 48 ± 2.1% for the PBS group, with no significant difference observed between the two. In contrast, the platelet aggregation rate in the MPB‐NO‐UK@PM + NIR group significantly decreased to 31.7 ± 1.8%, demonstrating that the NO generated by the combination of MPB‐NO‐UK@PM and NIR can effectively inhibit platelet activation and aggregation. Additionally, the scanning electron microscopy results (Figure 4E) provided further visual confirmation of these findings.

### 2.8 H₂O₂ Scavenging Capacity in Cells and Apoptosis Rate of Cells

In an H₂O₂‐induced HUVEC model, we assessed the intracellular antioxidant efficacy of MPB‐NO‐UK@PM using CLSM. Compared to the control group treated solely with H₂O₂, we observed a significant reduction in intracellular ROS levels in cells treated with MPB‐NO and MPB‐NO‐UK@PM (Figure 4F,G). These results demonstrate that MPB‐NO‐UK@PM, through the nanozyme activity of MPB‐NO, can effectively decrease intracellular ROS levels. Additionally, in the H₂O₂‐induced HUVEC model, we evaluated the cell apoptosis rate using flow cytometry. Compared to the untreated blank control, the apoptosis rate was significantly increased in cells treated with H₂O₂; however, the addition of MPB‐NO and MPB‐NO‐UK@PM nanoparticles effectively inhibited apoptosis in HUVECs (Figure S8, Surporting Informtion).

### 2.9 Biosafety In Vitro and In Vivo

**Figure 5:**
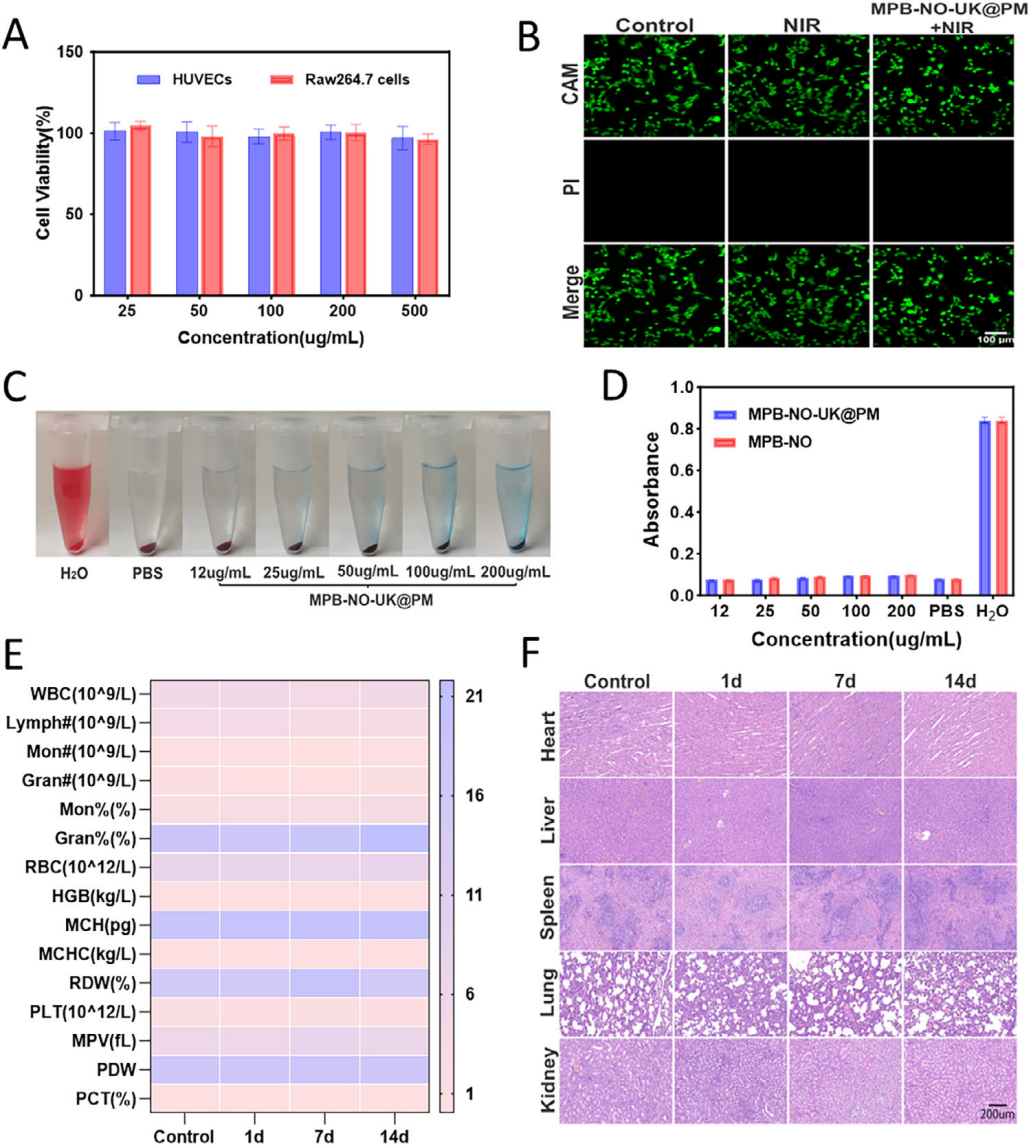
Biosafety in vitro and in vivo. A) Relative cell viabilities of RAW264.7 cells and HUVECs after coincubation with MPB‐NO‐UK@PM (*n* = 3). B) CLSM images of live and dead HUVECs after NIR irradiation. C) Images of red blood cells after co‐incubation with MPB‐NO‐UK@PM at various concentrations. D) The absorbance at 540 nm of blood samples incubated with various concentrations of MPB‐NO‐UK@PM and MPB‐NO. PBS and ultrapure water were used as negative and positive controls, respectively (*n* = 3). E) Blood routine of SD rats (*n* = 3). F) H&E staining of major organs of SD rats.

The safety of nanomaterials in biomedical applications has been a significant concern. Initially, we assessed cell viability using the Cell Counting Kit‐8 (CCK‐8) assay, which revealed no significant cytotoxicity when HUVECs and RAW264.7 cells were co‐cultured with various concentrations of MPB‐NO‐UK@PM (**Figure** **5**A). Subsequently, we evaluated the damage to HUVECs induced by photothermal therapy through a live/dead cell double staining method, and the results indicated no abnormal cell conditions (Figure 5B). Furthermore, to assess the hemocompatibility of the nanoparticles, we conducted a hemolysis experiment. In comparison to the negative control treated with PBS, diluted red blood cells incubated with different concentrations of MPB‐NO‐UK@PM and MPB‐NO appeared as clear solutions after centrifugation (Figure 5C; Figure S9, Supporting Information) and did not exhibit significant changes in absorbance values (Figure 5D). These findings suggest that the red blood cells co‐incubated with the nanoparticles maintained their structural integrity, with no evidence of hemolysis or hemoglobin release. Therefore, it can be concluded that both the unloaded MPB‐NO nanoparticles and the drug‐loaded enveloped MPB‐NO‐UK@PM nanocomposites demonstrate excellent blood biocompatibility.

Following the administration of MPB‐NO‐UK@PM into Sprague‐Dawley (SD) rats, assessments were performed on days 1, 7, and 14. The results indicated no significant abnormal alterations in hematological parameters (Figure 5E), blood biochemical parameters (Figure S10, Supporting Information), or H&E‐stained sections of major organs, including the heart, liver, spleen, lungs, and kidneys (Figure 5F) when compared to the control group. Additionally, Figure S11 (Supporting Information) illustrates the trend in body weight of the rats over the 14 days post‐administration. Observations indicated that the body weight of rats in both groups increased over time. Notably, the MPB‐NO‐UK@PM‐treated group did not demonstrate significant differences in body weight compared to the saline group.

Thrombolytic drugs possess a broad action spectrum, systematically activating plasminogen in the bloodstream, which subsequently increases plasmin activity and enhances the propensity for bleeding. To mitigate this risk, the nanocomposite delivery system developed in this study aims to minimize bleeding side effects through the targeted delivery of thrombolytic drugs. As illustrated in Figure S12 (Supporting Information), tail bleeding times in the MPB‐NO‐UK@PM group (5.31 min) and the MPB‐NO‐UK group (6.35 min) were prolonged compared to the control groups receiving saline (3.43 min) and MPB‐NO (3.65 min). However, these times were shorter than those observed in the free UK group (8.86 min). Notably, the MPB‐NO‐UK@PM group, which is coated with a platelet membrane to prevent the premature leakage of UK, demonstrated superior efficacy in reducing the risk of bleeding compared to the MPB‐NO‐UK group.

### 2.10 Targeting Ability of MPB‐NO‐UK@PM In Vivo and In Vitro

**Figure 6:**
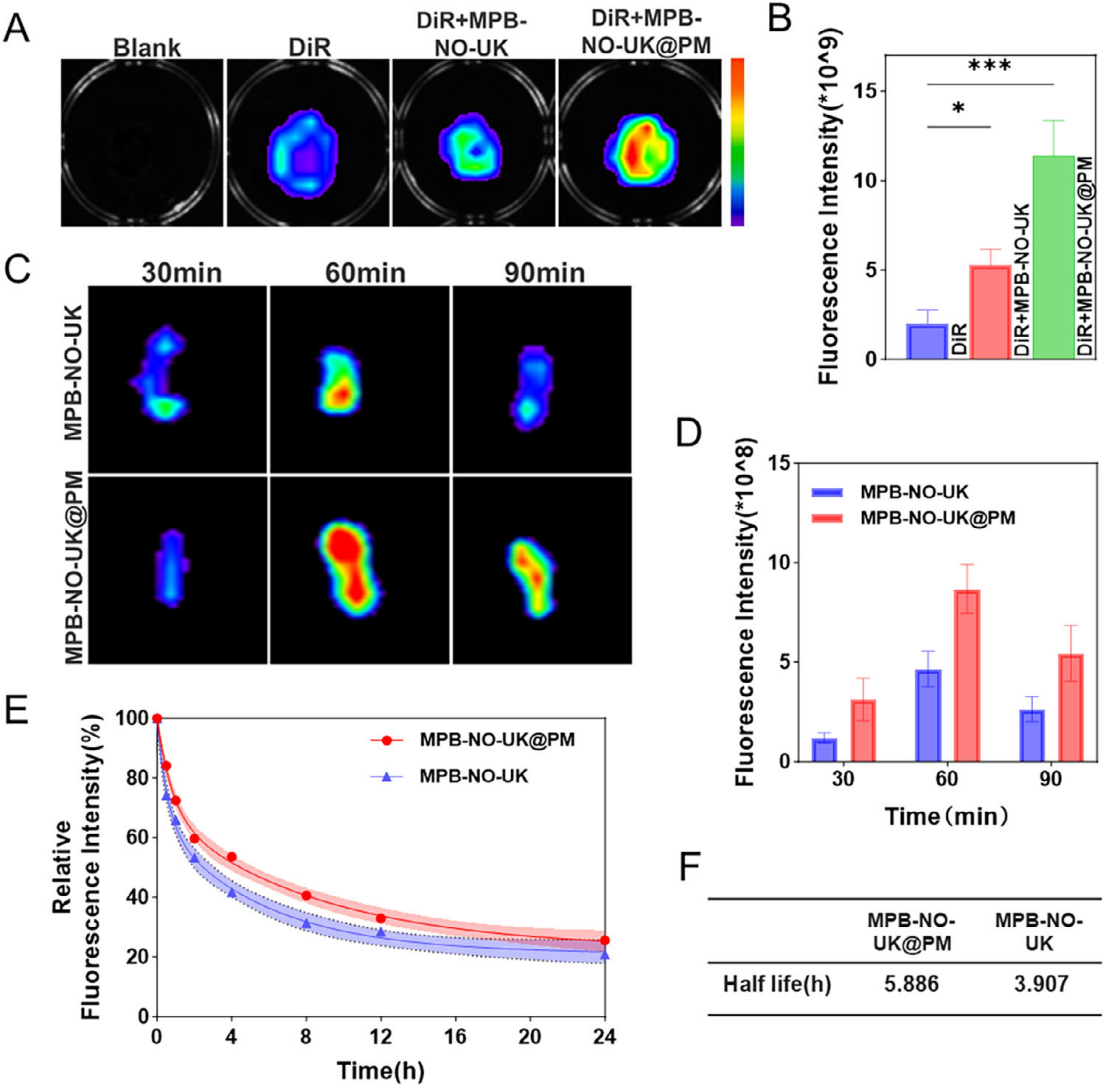
Targeting ability in thrombi and distribution in vivo of MPB‐NO‐UK@PM. A) Fluorescence images and intensity of platelet‐rich clot after different treatments. B) Quantitative analysis of fluorescence intensity of the platelet‐rich clot (*n* = 3). C) Fluorescence images of carotid artery thrombus at different times. D) Quantitative analysis of fluorescence intensity of the carotid artery thrombus (*n* = 3). E,F) The half‐life of MPB‐NO‐UK@PM and MPB‐NO‐UK. *P < 0.05, ***P < 0.001.

In an in vitro environment, platelet‐rich plasma rapidly transforms into a thrombus upon activation by thrombin and CaCl_2_. Subsequently, we co‐incubated DiR and DiR‐labeled MPB‐NO‐UK or MPB‐NO‐UK@PM with these platelet‐rich thrombi to investigate the specific binding ability of these nanoparticles to the thrombi. As illustrated in **Figure** **6**A,B, the fluorescence of the thrombi in the MPB‐NO‐UK group was slightly enhanced compared to the weak fluorescent signal observed in the DiR group, which can be primarily attributed to the non‐specific adsorption of nanoparticles on the thrombus surface. Notably, the thrombi in the MPB‐NO‐UK@PM group exhibited a significantly increased fluorescent intensity, indicating that MPB‐NO‐UK@PM can achieve efficient and specific accumulation in platelet‐rich thrombi due to the specific proteins present on the platelet membrane.

Next, we further investigated the in vivo targeting efficacy of the nanocomposites using a FeCl_3_‐induced carotid artery thrombosis model in SD rats. Following intravenous administration of MPB‐NO‐UK or MPB‐NO‐UK@PM, the carotid arteries were isolated at predetermined time points and subjected to ex vivo fluorescent imaging analysis. The results indicated that the fluorescent intensity of the carotid artery thrombi treated with both nano‐medicines peaked at 60 min post‐injection, followed by a gradual decrease in fluorescence intensity at 90 min. Notably, the fluorescent signal in the MPB‐NO‐UK@PM group was significantly stronger than that in the MPB‐NO‐UK group at all examined time points (Figure 6C,D). This finding suggests that encapsulation with platelet membranes enhances the targeting performance of MPB‐NO‐UK@PM toward arterial thrombi.

### 2.11 Distribution and Metabolism of MPB‐NO‐UK@PM In Vivo

Studying the distribution and metabolism of nanomedicines in animals is crucial for enhancing drug targeting and stability, as well as for evaluating drug safety and efficacy. During a 90‐minute circulation period, fluorescent imaging of rat organs was conducted, revealing that nanoparticles from both the MPB‐NO‐UK and MPB‐NO‐UK@PM groups exhibited varying degrees of fluorescent labeling in the liver, spleen, and lungs (Figure S13a, Supporting Information). However, imaging performed 24 h later demonstrated a significant reduction in fluorescent intensity in the liver region, with fluorescent signals in the spleen and lungs nearly completely dissipated (Figure S13b, Supporting Information). This phenomenon suggests that the nanocomposites do not accumulate long‐term in various organ tissues but are effectively eliminated and excreted by the body. Upon injection into the bloodstream, UK is rapidly inactivated by plasminogen activator inhibitor in the plasma and metabolized by the liver before excretion, exhibiting an elimination half‐life of ≈0.4 h.^[^ ^42^ ^]^ As illustrated in Figure 6E,F, the blood half‐lives of MPB‐NO‐UK and MPB‐NO‐UK@PM in rats were found to be 3.907 and 5.886 h, respectively. This finding indicates that utilizing nanocarriers for drug delivery can significantly enhance the circulation time of UK within the body, and the encapsulation with platelet membranes further extends the blood half‐life of the nanoparticles.

### 2.12 Photothermal Effect and Photoacoustic Imaging of MPB‐NO‐UK@PM In Vivo

**Figure 7:**
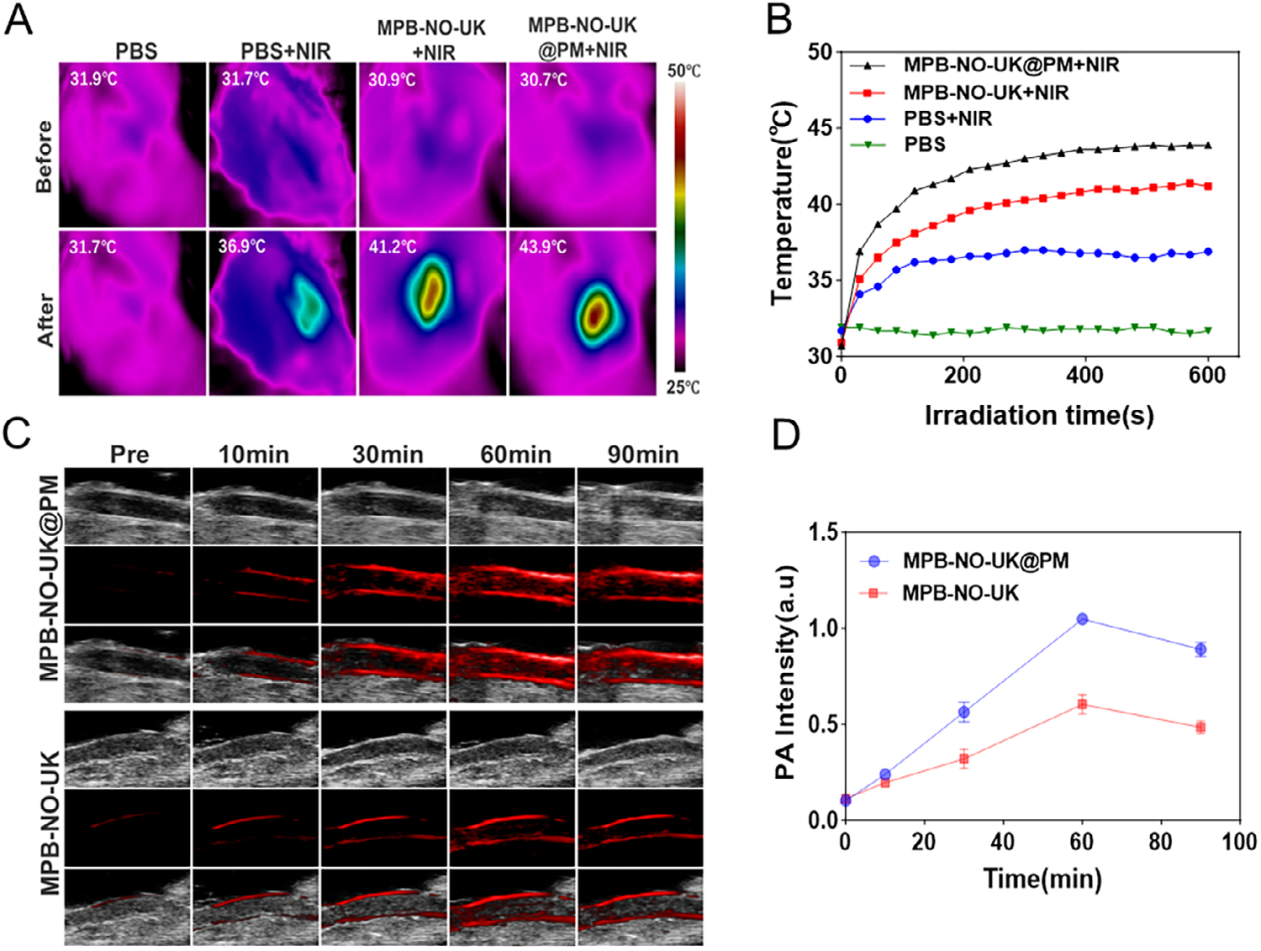
Photothermal Effect and Photoacoustic Imaging of MPB‐NO‐UK@PM in vivo. A) Thermal images of right carotid artery thrombus after NIR irradiation. B) Corresponding temperature variation curves after NIR irradiation. C) Photoacoustic images of the right carotid artery thrombus at different times. D) Quantitative analysis of PA intensity at different times (*n* = 3).

The exceptional photothermal effect of the nanocomposites was crucial to this study, particularly in applications such as photothermal thrombolysis and the promotion of NO and UK release. Consequently, we further assessed the photothermal effect of the nanocomposites in a rat model. As illustrated in **Figure** **7**A,B, under the irradiation of an 808 nm laser, the temperature in the thrombus region significantly increased in both the MPB‐NO‐UK and MPB‐NO‐UK@PM groups compared to the PBS control group and the laser‐only group. Furthermore, the temperature increase in the MPB‐NO‐UK@PM group was more pronounced than that observed in the MPB‐NO‐UK group.

Starting 10 min after the injection of the nanocomposite into rats, the photoacoustic (PA) imaging system detected PA signals in the thrombus region of the right carotid artery for both the targeted group (MPB‐NO‐UK@PM) and the non‐targeted group (MPB‐NO‐UK). Notably, the signal intensity in the targeted group was significantly higher than that in the non‐targeted group (Figure 7C). Further quantitative analysis of the PA signals revealed that both groups reached their peak PA signal intensity at 60 min post‐injection, with a subsequent decreasing trend in signal intensity observed by 90 min (Figure 7D).

### 2.13 Thrombolysis Therapy In Vivo

**Figure 8:**
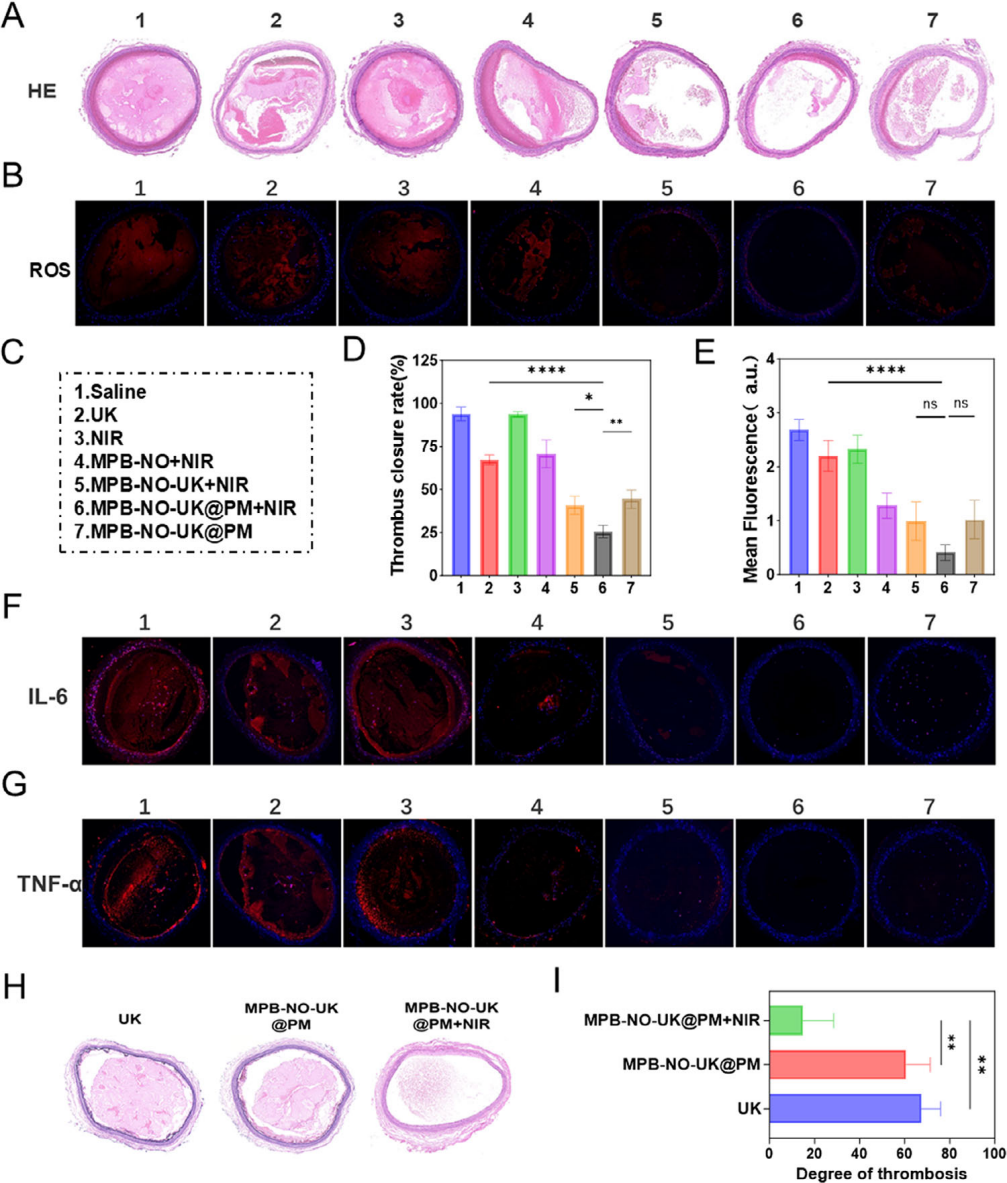
Thrombolysis therapy and prevention in vivo. A) H&E staining of artety carotid thrombi 24 h after corresponding treatment. B) DHE staining of artety carotid thrombi after corresponding treatment. C) The grouping of arterial thrombus treatment. D) Quantitative analysis of thrombus closure rate after corresponding treatment (*n* = 3). E) Quantitative analysis of ROS after corresponding treatment (*n* = 3). F) Immunofluorescence staining of (IL‐6) in the carotid artery after corresponding treatment. G) Immunofluorescence staining of (TNF‐α) in the carotid artery after corresponding treatment. H) HE staining of the carotid artery after the thrombosis prevention experiment. I) Quantitative analysis of thrombus area after the thrombosis prevention experiment (*n* = 3). *P < 0.05, **P < 0.01, ****P < 0.0001, n.s.: no significance. [Correction added on 18 April 2025, after first online publication: Figure 8 is updated with the revised version.]

To evaluate the thrombolytic efficacy of the nanocomposite in vivo, we conducted thrombolytic therapy using a carotid artery thrombosis model in SD rats. As illustrated in **Figure** **8**C, the experiment was divided into seven groups: Saline, UK, NIR, MPB‐NO+NIR, MPB‐NO‐UK+NIR, MPB‐NO‐UK@PM+NIR, and MPB‐NO‐UK@PM. Twenty‐four hours after the corresponding treatments, the affected carotid arteries underwent H&E staining, and the residual thrombus area within the lumen was quantitatively analyzed. As shown in Figure 8A,D, significant thrombus remained in the Saline and NIR groups, with residual areas of 93.9 ± 4% and 93.9 ± 1.5%, respectively. The UK and MPB‐NO+NIR groups exhibited moderate thrombus dissolution, with residual thrombus areas of 67.2 ± 2.9% and 70.7 ± 8%, respectively. Notably, in the MPB‐NO‐UK+NIR and MPB‐NO‐UK@PM groups, the thrombus areas were markedly reduced, with residual areas of 40.7 ± 5.2% and 44.4 ± 5.4%, respectively. Importantly, the MPB‐NO‐UK@PM group combined with NIR treatment demonstrated near‐complete thrombus dissolution, with a residual proportion of only 25.6 ± 3.7%. This result was significantly superior to all other groups, highlighting the excellent targeting capability of the nanocomposite and its synergistic photothermal/pharmacological thrombolytic effect.

In addition to H&E staining, we conducted DHE staining and immunofluorescence staining for TNF‐α and IL‐6 on the treated arterial thrombi to evaluate the antioxidant and anti‐inflammatory effects of the nanodrug. Strong DHE fluorescence was observed in the carotid artery thrombi and the vascular endothelial regions of the Saline, UK, and NIR groups, indicating a high presence of ROS at the thrombus site. In contrast, due to the nanozyme activity within the nanocomposite, only minimal DHE fluorescence was detected in the MPB‐NO+NIR, MPB‐NO‐UK+NIR, MPB‐NO‐UK@PM+NIR, and MPB‐NO‐UK@PM groups, suggesting effective ROS scavenging (Figure 8B,E). Concerning the anti‐inflammatory effects, the MPB‐NO+NIR, MPB‐NO‐UK+NIR, MPB‐NO‐UK@PM+NIR, and MPB‐NO‐UK@PM groups exhibited significantly reduced expression levels of IL‐6 and TNF‐α compared to the elevated expression levels found in the Saline, UK, and NIR groups (Figure 8F,G).

### 2.14 Thrombosis Prevention In Vivo

To evaluate the inhibitory effect of NO released from MPB‐NO‐UK@PM under NIR excitation on thrombosis, a rat carotid artery thrombosis model was employed, accompanied by H&E staining for verification. As illustrated in Figure 8H, significant thrombus formation was observed in the carotid artery following the injection of either UK or MPB‐NO‐UK@PM alone. In contrast, only a minimal amount of thrombus was detected in the MPB‐NO‐UK@PM combined with the NIR irradiation group. Further quantitative analysis of the thrombus area indicated that the UK group and the MPB‐NO‐UK@PM group exhibited thrombus areas of 67.4 ± 8.6% and 60.71 ± 10.6%, respectively, while the thrombus area in the MPB‐NO‐UK@PM + NIR group was significantly reduced to 14.81 ± 13.7% (Figure 8I), demonstrating a markedly superior antithrombotic effect compared to the other groups. Although the UK is a first‐line thrombolytic agent in clinical practice, it did not effectively prevent thrombus formation. In comparison to the MPB‐NO‐UK@PM group without NIR stimulation, the MPB‐NO‐UK@PM + NIR group exhibited significant antithrombotic efficacy, indicating that NO released from MPB‐NO‐UK@PM when induced by NIR can effectively reduce platelet adhesion and aggregation, thereby preventing thrombus re‐aggregation.

To monitor the recurrence of thrombosis following treatment, rats in the MPB‐NO‐UK@PM+NIR group underwent H&E staining of the carotid artery thrombus seven days post‐treatment. Spectral Doppler images of the right carotid artery were recorded using an ultrasound imaging system at three‐time points: before thrombosis formation, after thrombosis formation, and seven days post‐treatment. The results indicated that H&E staining showed no significant increase in the thrombus area seven days after treatment (Figure S14, Supporting Information). Furthermore, the ultrasound images revealed that no arterial spectral Doppler signal was detected at the thrombus site following its formation; however, the signal nearly returned to pre‐thrombosis levels seven days post‐treatment (Figure S15, Supporting Information). These findings demonstrate that the combined MPB‐NO‐UK@PM and NIR treatment not only exhibits excellent thrombolytic efficacy but also effectively prevents thrombus recurrence.

## 3 Conclusion

In this study, we developed mechanical biomimetic nanocomposites (MPB‐NO‐UK@PM) aimed at enhancing thrombolysis and preventing recurrence, based on the characteristics of the biological microenvironment associated with arterial thrombosis, which is characterized by high shear stress and an inflammatory environment rich in ROS. Following intravenous injection, the nanocomposite, aided by the platelet membrane, demonstrates a prolonged circulation time in vivo and effectively targets the site of arterial thrombosis. Upon NIR irradiation, the nano‐drug induces a localized temperature increase at the thrombus site, which triggers the release of UK and NO, thereby achieving a synergistic therapeutic effect through both photothermal and pharmacological thrombolysis. Concurrently, the released NO inhibits platelet adhesion and aggregation, while the nanozyme activity of Prussian blue scavenges ROS and inflammatory factors within the thrombus, thus enhancing the biological microenvironment and effectively preventing thrombus recurrence. Additionally, the nanocomposite facilitates the visualization of the thrombus via photoacoustic imaging. This study presents a comprehensive, efficient, and safe strategy for the management of arterial thrombosis.

## 4 Experimental Section

### Materials

Potassium ferricyanide (K_3_[Fe(CN)_6_]), sodium nitroprusside (Na_2_[Fe(CN)_5_NO]·2H_2_O), polyvinylpyrrolidone (PVP, K30), and TMB color developing solution were sourced from Aladdin Reagent Co., Ltd. (Shanghai, China). Urokinase was obtained from Macklin Biochemical Technology Co., Ltd. (Shanghai, China). The total nitric oxide assay kit, total superoxide dismutase (SOD) activity assay kit (WST‐8 method), Hoechst 33342, DCFH‐DA assay kit, and Fluo‐4 calcium ion assay kit were procured from Beyotime Biotechnology Co., Ltd. (Jiangsu, China). The UK ELISA kit was acquired from Boshide Bioengineering Co., Ltd. (Wuhan, China). The phospho‐eNOS(Ser1177) antibody was obtained from MedChemExpress (New Jersey, America).

### Preparation of MPB and MPB‐NO

To synthesize MPB nanoparticles, potassium ferricyanide(120 mg) and PVP(3 g) were dissolved in HCl(0.01 m, 40 mL) and stirred at room temperature for 30 min Subsequently, the mixture was heated to 80 °C for 1 h. MPB nanoparticles were then collected by centrifugation at 11 000 rpm for 10 min and washed three times with deionized water. For the synthesis of MPB‐NO nanoparticles, potassium ferricyanide (60 mg), SNP(488.7 mg), and PVP(3 g) were dissolved in HCl (0.1 m, 40 mL) and stirred at room temperature for 30 min. The mixture was then heated to 80 °C for 12 h. MPB‐NO nanoparticles were collected by centrifugation at 11 000 rpm for 10 min and subsequently washed three times with deionized water.

### Preparation of MPB‐NO‐UK@PM

To prepare MPB‐NO‐UK, UK (1 mg) and MPB‐NO (5 mg) were first dissolved in deionized water (5 mL) and stirred continuously at room temperature for 12 h. MPB‐NO‐UK was then obtained by centrifugation at 11 000 rpm for 10 min. Subsequently, the PM was extracted and prepared following the method described in the reference.^[^ ^43^ ^]^ Blood samples were collected from 8‐ to 10‐week‐old male SD rats and placed in anticoagulant tubes containing EDTA. The samples were then centrifuged at 4 °C at 1300 rpm for 20 min to separate the supernatant containing PRP. To inhibit platelet activation, the PRP was transferred to a PBS buffer solution containing prostaglandin E1. Next, the solution was centrifuged at 4 °C at 3500 rpm for 20 min to isolate the platelets, which were subsequently resuspended in a PBS solution containing protease inhibitors. PM was obtained by repeatedly freezing and thawing the platelet suspension at −80 °C and room temperature, respectively. Following this, PM was separated by centrifugation (8500 rpm, 5 min) and dispersed in ultrapure water. Finally, the PM suspension was sonicated in an ice‐water bath using an ultrasonic oscillator (SONICS & MATERIALS, Inc., USA) at 60 W for 3 min, and then mixed with the MPB‐NO‐UK solution at a 1:1 mass ratio. The mixture was again sonicated at 60 W for 3 min and subsequently extruded through a liposome extruder (Avanti Polar Lipids, Inc., USA) to obtain MPB‐NO‐UK@PM.

### Characterization

The morphologies of MPB‐NO and MPB‐NO‐UK@PM were observed using transmission electron microscopy (TEM, FEI Tecnai G2 12, FEI Co., USA). To further investigate the internal structure and elemental distribution of MPB‐NO, high‐resolution transmission electron microscopy (HRTEM, FEI Tecnai G2 F30, USA) was employed. The size and zeta potential of the nanoparticles, as well as the size stability of MPB‐NO‐UK@PM after 1, 3, and 7 days of preparation, were determined using a dynamic light scattering laser particle size analyzer (Zetasizer Nano ZS90, Malvern Instruments Ltd., Worcs, UK) at 25 °C. The structures of MPB and MPB‐NO were studied using Fourier transform infrared spectroscopy (FTIR, Thermo Scientific Nicolet IS10, USA), X‐ray photoelectron spectroscopy (XPS, Thermo Escalab 250XI, USA), and X‐ray diffraction (XRD, BRUKER D8 ADVANCE, Germany). The specific surface area of MPB‐NO was measured with a specific surface area analyzer (BET, Quantachrome NOVA Touch, USA). The free UK concentration in the MPB‐NO‐UK supernatant was evaluated utilizing an ELISA kit. The calculations for encapsulation efficiency and drug loading efficiency were performed as follows: Encapsulation efficiency (%) = [(*W*_t_ – *W*_f_)/*W*_t_] × 100%; Drug loading efficiency (%) = [(*W*_t_ – *W*_f_)/*W*_p_] × 100%, where *W*_f_ denotes the free drug content, W_t_ represents the total drug amount added, and *W*_p_ indicates the total amount of carrier plus drug. Verification of the PM and MPB‐NO‐UK@PM was conducted using sodium dodecyl sulfate‐polyacrylamide gel electrophoresis (SDS‐PAGE).

### Photothermal Effect In Vitro

Solutions of MPB (100 µg mL⁻^1^), MPB‐NO (100 µg  mL⁻^1^), and PBS were separately exposed to an 808 nm laser (1 W cm⁻^2^, 10 min). MPB‐NO‐UK@PM at varying concentrations (200, 100, 50, 25, and 12.5 µg mL⁻^1^) were irradiated under the same conditions. MPB‐NO‐UK@PM(100 µg mL⁻^1^) was treated with an 808 nm laser at different power densities (0.5, 0.8, 1, and 1.5 W cm⁻^2^). To assess the photothermal stability, MPB‐NO‐UK@PM (100 µg mL⁻^1^) underwent four cycles of on/off 808 nm laser (1 W cm⁻^2^) irradiation. Throughout all experiments, an infrared thermal imaging camera was utilized to track temperature variations.

### Photoacoustic Imaging In Vitro

The optimal photoacoustic excitation wavelength for MPB‐NO‐UK@PM within the wavelength range of 680–930 nm was first determined. MPB‐NO‐UK@PM was prepared at various concentrations (25, 50, 100, 200, and 400 µg mL⁻^1^). A 100 µL aliquot of each solution was added to the wells of a biomimetic gel. Subsequently, the photoacoustic imaging system (VisualSonics Inc., Toronto, Canada) was employed to scan the samples, and the resulting photoacoustic signals were quantitatively analyzed.

### NIR‐Responsive Release of NO In Vitro

To investigate the NO release behavior of SNP, MPB, and MPB‐NO under NIR irradiation, respective suspensions (1 mg mL⁻^1^, 1 mL) were irradiated with an 808 nm laser (1 W cm⁻^2^). Samples of the supernatant were taken at 0, 5, 10, 15, and 20 min of irradiation, and the NO content was measured using a Total Nitric Oxide Assay Kit. Specifically, MPB‐NO‐UK@PM (1 mg mL ⁻^1^) was subjected to irradiation with an 808 nm laser at power densities of 0, 0.8, 1, and 1.5 W. Samples were collected at irradiation times of 0, 5, 10, 15, and 20 min. The NO content in the supernatants was measured.

### NIR‐Responsive Release of UK In Vitro

The MPB‐NO‐UK@PM solution was placed in a sealed dialysis bag (MWCO = 100 kDa) and immersed in 50 mL of phosphate‐buffered saline (PBS). The sample was incubated at 37 °C with shaking at 100 rpm. Subsequently, the MPB‐NO‐UK@PM solution was irradiated with an 808 nm laser (1 W cm⁻^2^, 10 min), while a control group remained unirradiated. At various time points (0, 0.25, 0.5, 1, 2, 3, 6, 12, and 24 h), 100 µL of the solution was withdrawn, appropriately diluted, and assayed using a UK ELISA kit.

### ROS Scavenging Properties In Vitro

The POD activity of MPB‐NO‐UK@PM was assessed using the TMB chromogenic substrate. MPB‐NO‐UK@PM (100 µg mL⁻¹, 50 mL) was mixed with TMB chromogenic substrate (100 µL) and allowed to react at room temperature, with PBS serving as a control. Immediately after initiating the reaction, absorbance at 650 nm was measured every minute for 10 min. For CAT activity, MPB‐NO‐UK@PM (1 mg mL⁻¹, 300 µL) and H₂O₂ (30%, 3 mL) were combined with PBS (pH 7.2–7.4, 25 mL), and oxygen concentration was monitored for 10 min using a portable dissolved oxygen meter. SOD activity was determined using a Total SOD Activity Assay Kit (WST‐8 method). MPB‐NO‐UK@PM was prepared at various concentrations (25, 50, 100, 200, and 400 µg mL⁻¹), and absorbance at 450 nm was measured after incubation with the detection reagent at 37 °C for 30 min. Percentage inhibition and enzyme activity units were calculated according to the kit instructions.

### Cell Lines and Animals

HUVECs and RAW264.7 cells were obtained from the Chongqing Key Laboratory of Ultrasound Molecular Imaging. Experimental animals were acquired from the Experimental Animal Center of Chongqing Medical University. All animal experiments were performed in accordance with the guidelines set forth by the Animal Ethics Committee of Chongqing Medical University, and the protocols received approval from the Committee for the Management and Use of Experimental Animals at Chongqing Medical University (Ethics Approval Number: IACUC‐CQMU‐2024‐0637).

### Effect of Shear Force on Piezo1 Activation

HUVECs were seeded onto glass slides and cultured in a humidified environment at 37 °C with 5% CO_2_ for 24 h. A dynamic cell co‐culture system (Naturethink, NK110‐GPY, China) was employed to apply various shear stresses (3, 15, 30, and 45 dyne cm⁻^2^) to the HUVECs on the slides. Following co‐incubating the cells with fluo‐4AM, the levels of intracellular calcium ions were quantified using a flow cytometer. After the same treatment, the fluorescence intensity of HUVECs on the glass slides in each group was assessed using an inverted fluorescence microscope. After applying the corresponding shear stress to the endothelial cells, the cells were incubated overnight with the phospho‐eNOS (Ser1177) antibody, followed by a 1‐hour incubation with the secondary antibody labeled with FITC. Finally, the samples were observed under a confocal microscope.

### Intracellular Uptake

RAW 264.7 cells were seeded into confocal dishes and cultured for 24 h. DiI‐labeled MPB‐NO‐UK and MPB‐NO‐UK@PM (100 µg mL⁻^1^) were then added to the confocal dishes and the cells were further incubated for 4 h. The cell nuclei were stained with Hoechst 33342 staining solution for 20 min. Finally, the cells were observed using a laser confocal microscope (CLSM, Dragonfly 200, Andor, UK) and analyzed using Image J software.

### Targeting Specificity to Activated Platelets

HUVECs were seeded into confocal dishes and cultured for 24 h, after which platelets prelabeled with DiI were added. The platelets were activated with CaCl_2_ (0.1 µg) and thrombin (1 U) for 30 min, following which unactivated platelets were removed. Subsequently, FITC‐labeled MPB‐NO‐UK and MPB‐NO‐UK@PM (100 µg mL^−1^) were introduced to the cells and cultured for an additional 2 h. Excess nanoparticles were then washed away. The cell nuclei were stained using Hoechst 33342 staining solution for 20 min. Images were captured with a laser confocal microscope and analyzed using Image J software.

### Adhesion Characteristics to HUVECs

HUVECs were seeded into confocal dishes and cultured in an incubator for 24 h. Subsequently, the cells were co‐incubated with a medium containing H_2_O_2_ (0.5 mm) for 2 h to induce injury, while normal endothelial cells (without H_2_O_2_) served as the control group. DiI‐labeled MPB‐NO‐UK and MPB‐NO‐UK@PM (100 µg mL⁻^1^) were then added to both groups and incubated for an additional 2 h. Following this, cell nuclei were stained using Hoechst 33342 staining solution. Finally, the cells were observed with a laser confocal microscope, and the images were analyzed using ImageJ software.

### Assessment of Anti‐Platelet Aggregation Capability

PRP was prepared as previously described, and three different groups (*n* = 3) were established: PBS, MPB‐NO‐UK@PM, and MPB‐NO‐UK@PM+NIR. PRP (100 µL) was combined with an equal volume of either PBS or MPB‐NO‐UK@PM (100 µg mL⁻^1^) according to the group assignments. In the MPB‐NO‐UK@PM+NIR group, the solution was irradiated with an 808 nm laser (1 W cm⁻^2^, 10 min). Following this, the optical density (OD) values of each group were measured using a microplate reader at a wavelength of 490 nm. Subsequently, ADP (10 µmol L⁻^1^, 10 µL) was added to each well, and after shaking for 15 min, the OD values were measured again with the microplate reader. The platelet aggregation rate for each group was calculated using the formula: aggregation% = (1 – OD before ADP addition / OD after ADP addition) × 100%. Platelet aggregation in each group was further examined using a scanning electron microscope (SEM, SU8020, Hitachi, Japan).

### H₂O₂ Scavenging Capacity in Cells

HUVECs were seeded into confocal dishes and cultured for 24 h. Following this incubation period, the cells were treated with the following reagents for co‐incubation over 4 h: H_2_O_2_ (200 µm), H_2_O_2_ (200 µm) combined with MPB‐NO (100 µg mL⁻^1^), and H_2_O_2_ (200 µm) combined with MPB‐NO‐UK@PM (100 µg mL⁻^1^). Normal cells were utilized as the negative control. Subsequently, the cells were incubated with DCFH‐DA (10 µm) for 30 min, followed by 20‐minute staining of the nuclei with Hoechst 33342. Finally, the intracellular fluorescence intensity was observed using a laser confocal microscope to evaluate the levels of ROS.

### Apoptosis Rate of Cells

HUVECs were seeded into 6‐well plates and cultured for 24 h. The cells were divided into four groups: blank control, H_2_O_2_ (400 µm), H_2_O_2_ (400 µm) + MPB‐NO (100 µg mL⁻^1^), and H_2_O_2_ (400 µm) + MPB‐NO‐UK@PM (100 µg mL⁻^1^). After the aforementioned treatments, the cells were incubated for an additional 4 h. Subsequently, Annexin V‐FITC (5 µL) and propidium iodide staining solution (10 µL) were added, and the mixture was gently agitated. Following a 20‐minute incubation at room temperature, the results were analyzed using a flow cytometer (BD FACSCalibur, Becton, Dickinson and Company, USA).

### Toxicity Evaluation of MPB‐NO‐UK@PM In Vitro

Logarithmically growing HUVECs and RAW264.7 cells were seeded into 96‐well plates and cultured for 24 h. MPB‐NO‐UK@PM at various concentrations (25, 50, 100, 200, and 500 µg mL⁻^1^) was added to the cells for an additional 12 h. Incubation utilizing 100 µL per well of a 10% CCK‐8 assay reagent was conducted for 45 min and cell viability was subsequently measured through a microplate reader to determine the absorbance at a wavelength of 450 nm.

### Photothermal Effect‐Induced Damage to Cells

HUVECs were seeded into confocal dishes and cultured for 24 h. MPB‐NO‐UK@PM solution (100 µg mL⁻^1^) was added to the corresponding groups. After 4 h of incubation, the respective groups were treated with an 808 nm laser (1 W cm⁻^2^, 10 min), and then further incubated for 2 h. Calcein AM/PI staining solution was added, and the cells were observed under a laser confocal microscope.

### Hemolysis Evaluation

Rat blood samples were collected in EDTA‐anticoagulated tubes and subjected to centrifugation (1300 rpm, 20 min) to obtain red blood cell pellets. MPB‐NO and MPB‐NO‐UK@PM at varying concentrations (12, 25, 50, 100, and 200 µg mL⁻^1^) were incubated with the red blood cells at 37 °C for 4 h. Following centrifugation (10 000 rpm, 5 min), the supernatants were collected for measurement of absorbance at 540 nm. Ultra‐pure water and PBS buffer were utilized as the positive and negative controls, respectively.

### Safety Evaluation of MPB‐NO‐UK@PM In Vivo

MPB‐NO‐UK@PM (1 mg mL⁻^1^,1 mL) and saline were injected into SD rats. Blood samples were collected from the orbital vein for routine blood tests and biochemical analysis on days 1, 7, and 14 post‐injection. Organ tissues were harvested for H&E staining. Throughout this period, the body weights of the rats were recorded every other day.

### Tail Bleeding Time Analysis

Rats were randomly divided into five groups (*n* = 3): saline, MPB‐NO, MPB‐NO‐UK, MPB‐NO‐UK@PM, and UK. The corresponding nanomedicines (1 mg mL⁻^1^, 1 mL) and UK (0.11 mg mL⁻^1^, 1 mL) were administered via tail vein injection. Twenty min after administration, the tails of the rats were amputated 1 cm from the tip and immediately immersed in a pre‐warmed PBS buffer solution at 37 °C. The duration of bleeding was monitored and recorded.

### In Vitro Targeting of MPB‐NO‐UK@PM

PRP was obtained and mixed with 10 µL of red blood cells. A mixture of the above solution (180 µL), thrombin (5 U), and CaCl_2_ (5 mmol L⁻^1^, 15 µL) was added to a 96‐well plate and incubated at 37 °C for 3 h to facilitate clot formation. The resulting clots were randomly divided into four groups (*n* = 3): saline, DiR, MPB‐NO‐UK+DiR, and MPB‐NO‐UK@PM+DiR. Each group was incubated with the respective preparations at 37 °C for 2 h, followed by washing with saline to remove any residuals. Imaging and quantitative analysis were performed using a multimodal in vivo animal imaging system (AniView100 Pro, Boluteng Biotechnology Co., Ltd., Guangzhou, China).

### Establishment of Carotid Artery Thrombosis Model

The right carotid artery of male SD rats (8–10 weeks, 250–320 g) was exposed and wrapped with filter paper soaked in FeCl_3_ (10%) for 10 min. Following this, the area was rinsed with saline. Throughout the procedure, the rats were maintained at a temperature of 37 °C on a heating pad.

### In Vivo Targeting of MPB‐NO‐UK@PM

Rats with established carotid artery thrombosis were randomly divided into two groups (*n* = 3): MPB‐NO‐UK and MPB‐NO‐UK@PM. DiR‐labeled MPB‐NO‐UK and MPB‐NO‐UK@PM (1 mg mL⁻^1^, 1 mL) were injected into the rats. The carotid artery thrombi were excised after 30, 60, and 90 min of nanoparticle circulation in the bloodstream. Fluorescence intensity was recorded using a multimodal in vivo imaging system. At 90 min and 24 h post‐injection, hearts, livers, spleens, lungs, and kidneys were collected for fluorescence imaging to evaluate the biodistribution of the nanoparticles.

### Half‐Life Measurement of MPB‐NO‐UK@PM

SD rats were divided into two groups (*n* = 3): MPB‐NO‐UK and MPB‐NO‐UK@PM. DiR‐labeled MPB‐NO‐UK and MPB‐NO‐UK@PM (1 mg mL⁻^1^, 1 mL) were injected into the rats. Blood samples (200 µL) were collected from the orbital venous plexus at 5 min, 0.5 h, 1 h, 2 h, 4 h, 8 h, 12 h, and 24 h post‐injection, and were added to Eppendorf tubes containing 4% sodium citrate (1:9). Following centrifugation (4000 rpm, 5 min), the supernatants were collected, diluted, and their fluorescence intensity was measured using a fluorescence spectrophotometer.

### Photothermal Effect In Vivo

SD rats with established carotid artery thrombosis models were randomly divided into four groups: saline, saline+NIR, MPB‐NO‐UK+NIR, and MPB‐NO‐UK@PM+NIR. Following the administration of the respective formulations to the rats, the thrombus site was irradiated with an 808 nm laser (1 W cm⁻^2^) for 10 min, and the temperature change at the thrombus site was recorded using a thermal infrared imaging camera.

### Photoacoustic Imaging In Vivo

Rats with established carotid artery thrombosis were intravenously injected with MPB‐NO‐UK and MPB‐NO‐UK@PM (1 mg mL⁻^1^, 1 mL) respectively. Photoacoustic images of the right carotid artery region were captured at various time points (0, 10, 30, 60, and 90 min) using a photoacoustic imaging system. A quantitative analysis of the photoacoustic signal intensity was conducted.

### Thrombolytic Therapy In Vivo

SD rats with right carotid artery thrombosis were randomly divided into seven groups (*n* = 3): saline, UK, NIR, MPB‐NO+NIR, MPB‐NO‐UK+NIR, MPB‐NO‐UK@PM+NIR, and MPB‐NO‐UK@PM. Corresponding nanomedicines (1 mg mL⁻^1^, 1 mL), saline (1 mL), or UK (0.11 mg mL^−1^, 1 mL) were then injected into the rats, followed by 808 nm laser(1 W cm⁻^2^, 10 min) irradiation. After 24 h, the SD rats were euthanized, and thrombi from the carotid arteries were harvested for H&E staining. The thrombus area was quantitatively analyzed using ImageJ software to evaluate thrombolytic efficacy. To assess the anti‐ROS effect of the nanomedicines at the thrombus site, the right carotid arteries were stained with DHE and subsequently imaged using a fluorescence microscope. To evaluate the anti‐inflammatory effect of the nanomedicines at the thrombus site, the right carotid arteries underwent immunofluorescence staining for IL‐6 and TNF‐α, followed by imaging with a fluorescence microscope.

### Evaluation of the Effectiveness of NO in Preventing Thrombosis

Healthy rats were randomly divided into three groups (n = 3): UK, MPB‐NO‐UK@PM, and MPB‐NO‐UK@PM+NIR. The right common carotid artery of each rat was exposed, and 1 mL of either UK (0.11 mg mL⁻^1^) or MPB‐NO‐UK@PM (1 mg mL⁻^1^) was injected. A filter paper soaked in FeCl_3_ (10%) was applied to the dorsal side of the carotid artery. Concurrently, the rats in the MPB‐NO‐UK@PM+NIR group received 808 nm laser (1 W cm⁻^2^, 10 min) irradiation on the ventral side of the carotid artery. The carotid artery segments were then harvested, stained with H&E, and the thrombus area was quantitatively analyzed using ImageJ software.

### Monitoring of Thrombus Recurrence

Three rats with established carotid artery thrombi were injected with MPB‐NO‐UK@PM (1 mg mL⁻^1^) and subsequently irradiated with an 808 nm laser (1 W cm⁻^2^, 10 min). Following a 7‐day period, the rats were euthanized, and the carotid artery thrombi were harvested for H&E staining. Spectral Doppler images of the right carotid artery were recorded using an ultrasound imaging system at three‐time points: before thrombus formation, after thrombus formation, and 7 days post‐treatment.

### Statistical Analysis

GraphPad Prism 10.1.2 (San Diego, CA, USA) was used for statistical analysis. All results were presented as the mean ± standard deviation derived from a minimum of three independent experiments. The *t‐*test was conducted to assess differences between the two groups. In scenarios where comparisons involved more than two groups, a one‐way ANOVA was employed, followed by post‐hoc *t*‐tests with Bonferroni correction for inter‐group comparisons. A significance level of *p* < 0.05 was established for determining statistically significant differences.

## Conflict of Interest

The authors declare no conflict of interest.

## Supporting information



Supporting Information

## Data Availability

The data that support the findings of this study are available in the supplementary material of this article.
