# Supplementary material for: Mechanical Biomimetic Nanocomposites for Multidimensional Treatment of Arterial Thrombosis
Source: Adv Sci (Weinh). 2025 Apr 15;12(25):2501134. doi: 10.1002/advs.202501134 (PMC12224924; doi:10.1002/advs.202501134)
Supplement: Supplementary file 1 — Supporting Information [file ADVS-12-2501134-s001.docx]

Supporting Information

**Mechanical Biomimetic Nanocomposites for Multidimensional Treatment of Arterial** **Thrombosis**

*Ying Li^#^, Qi Xiang^#^, Yan Zhang, Fuxue Luo, Yunfang Wu, Haitao Ran, and Yang Cao**

Chongqing Key Laboratory of Ultrasound Molecular Imaging and Therapy, Ultrasound Department of the Second Affiliated Hospital, Institute of Ultrasound Imaging, State Key Laboratory of Ultrasound in Medicine and Engineering, Chongqing Medical University, Chongqing 400016, China.

*Address correspondence to: yangcao@cqmu.edu.cn

**Figure S1.** XPS spectra of MPB and MPB-NO.


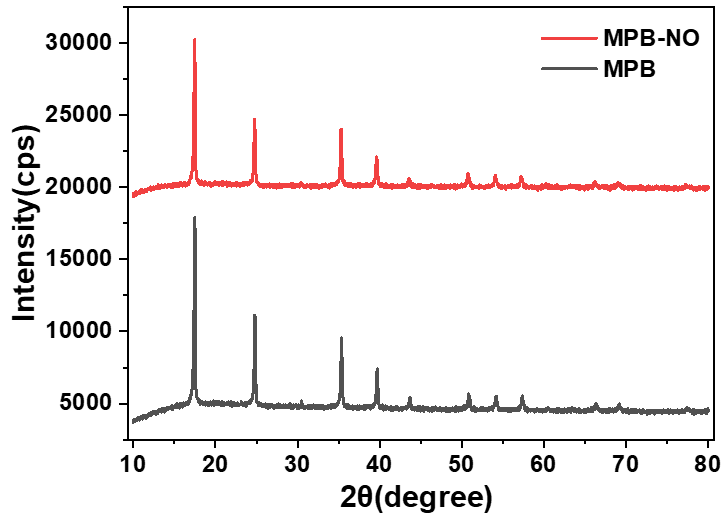


**Figure S2.** XRD patterns of MPB and MPB-NO.


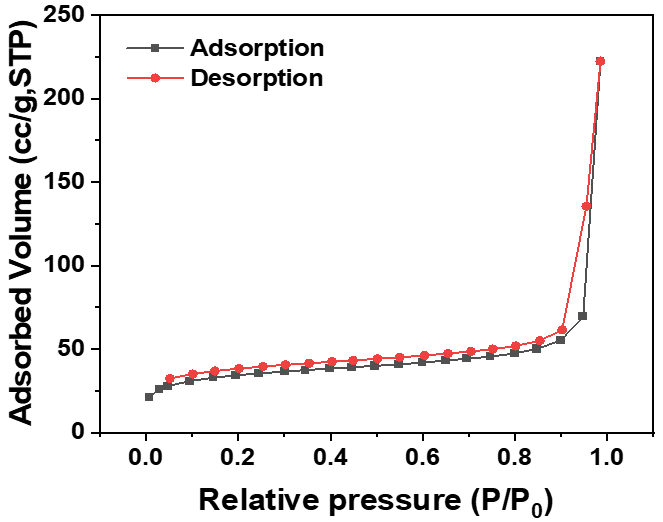


**Figure S3.** N_2_ adsorption-desorption isotherms of MPB-NO.


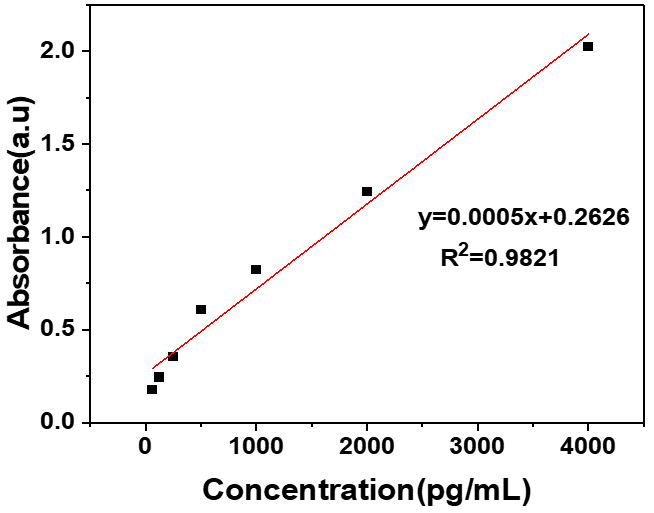


**Figure S4.** UK standard curve measured using the UK Elisa kit.

**Figure S5.** Temperature variation curves of MPB-NO-UK@PM after repeated exposure to 808 nm laser (1 W cm⁻^2^).


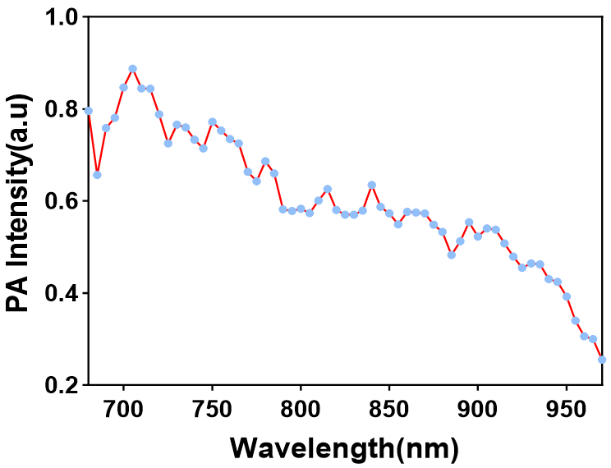


**Figure S6.** The variation of photoacoustic signals of MPB-NO-UK@PM within the wavelength range of 680-930 nanometers.


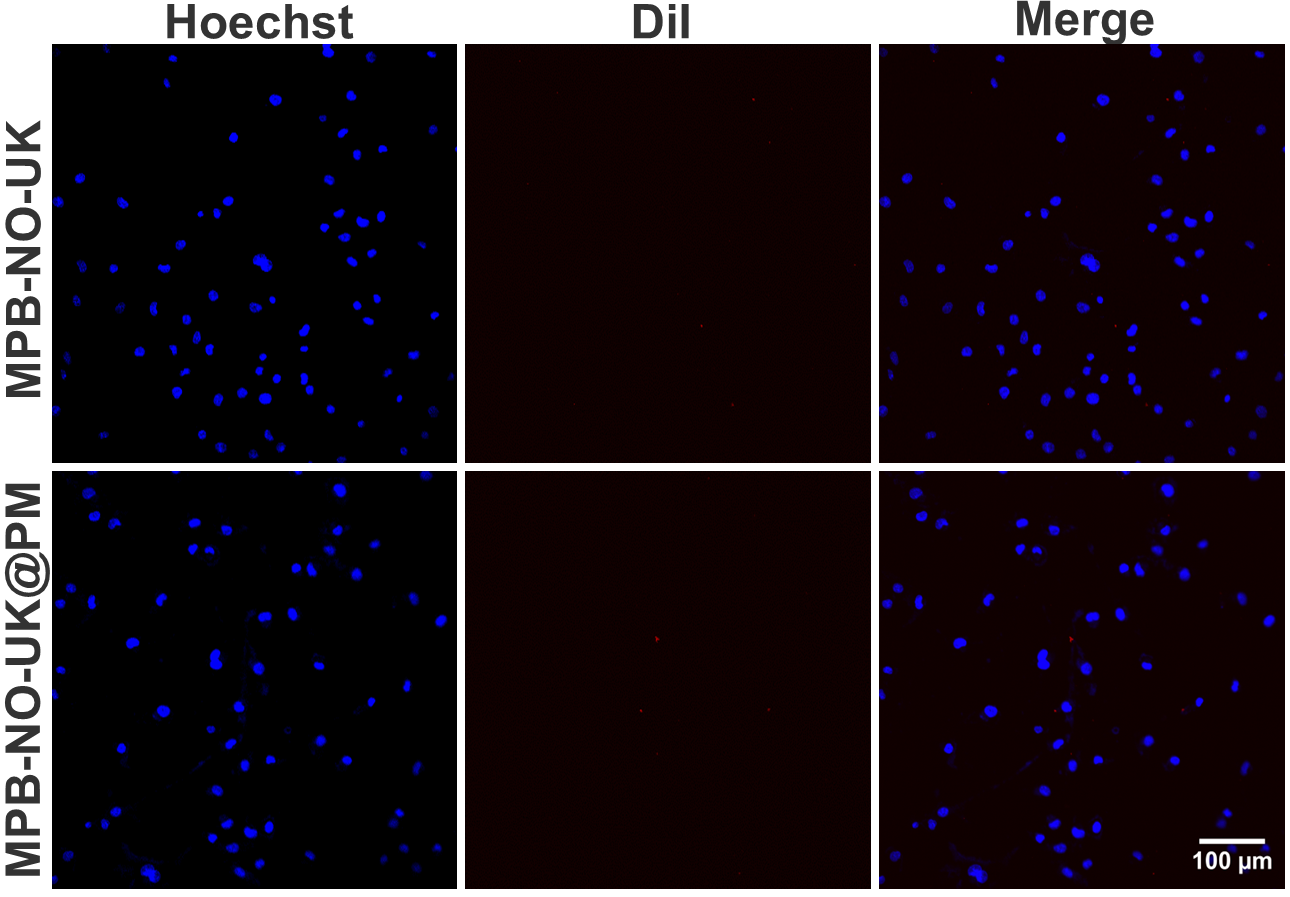


**Figure S7.** CLSM images of MPB-NO-UK and MPB-NO-UK@PM targeted to non-activated HUVECs.


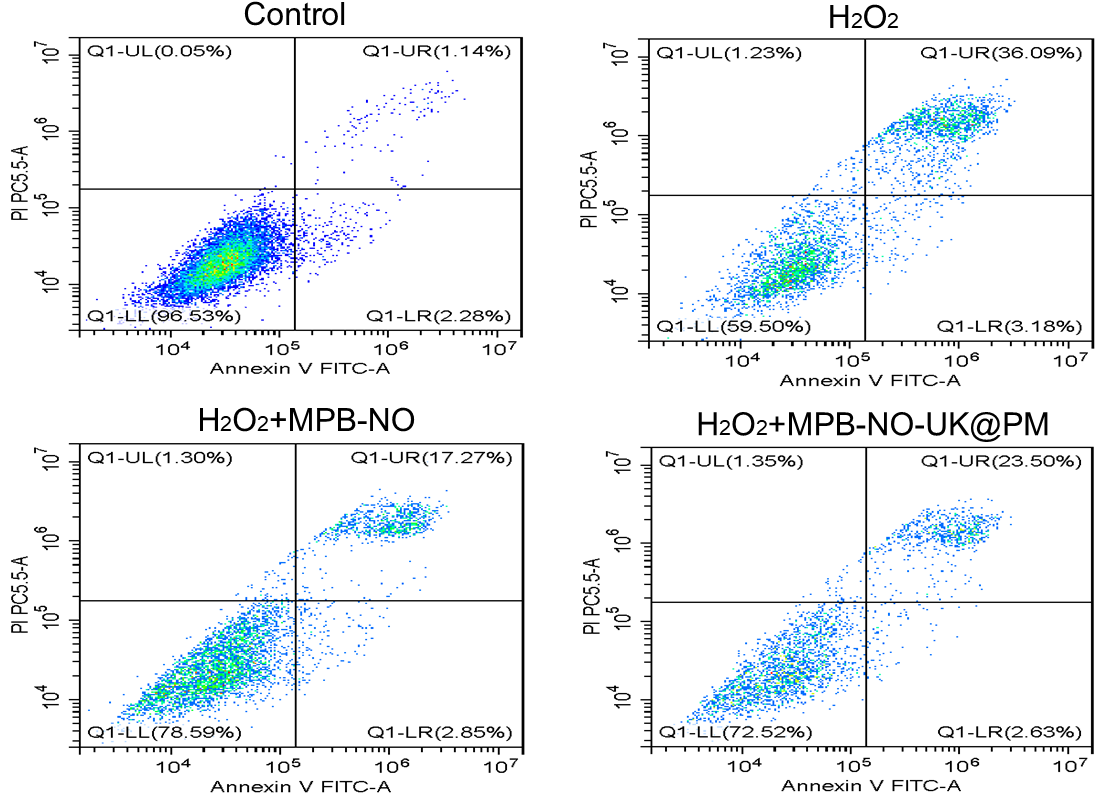


**Figure S8a.** Flow cytometry profiles of HUVECs after H₂O₂ stimulation and incubation with nanocomposite.

**Figure S8b.** Quantitative analysis of apoptosis rate of HUVECs after H₂O₂ stimulation and incubation with nanocomposites (*n* = 3). ***P < 0.001.


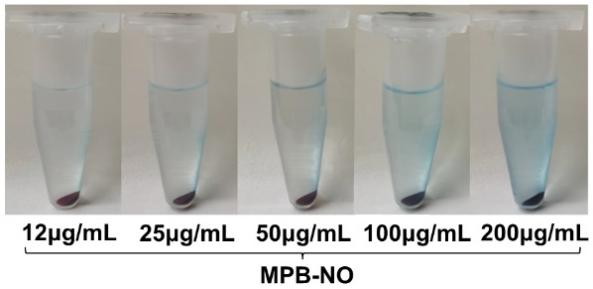


**Figure S9.** Images of red blood cells after co-incubation with MPB-NO at various concentrations.


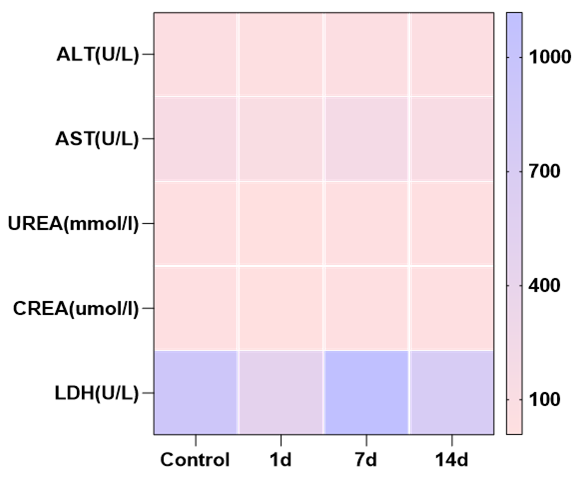


**Figure S10.** Biochemical markers relevant to hepatic and kidney functions in serum of SD rats (*n* = 3).


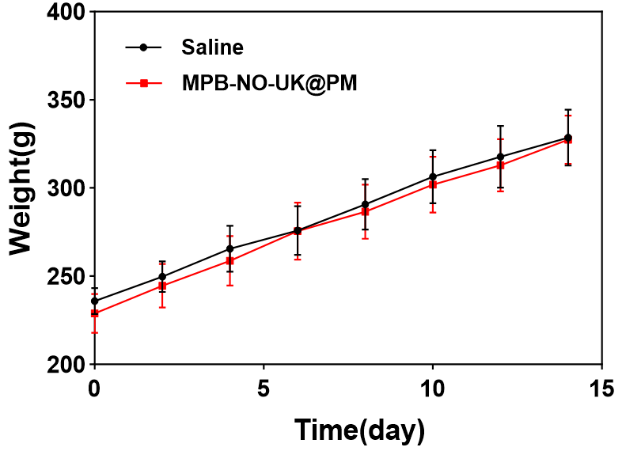


**Figure S11.** Body weight of the rats over the 14 days post-administration.


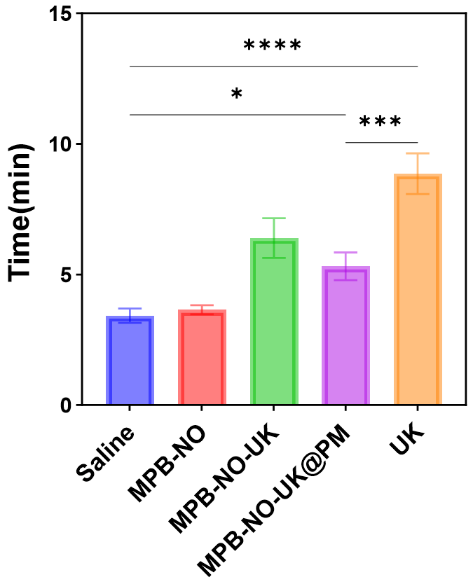


**Figure S12.** Tail bleeding times of rats after different treatments (*n* = 3). *P < 0.05, ***P < 0.001, ****P < 0.0001.


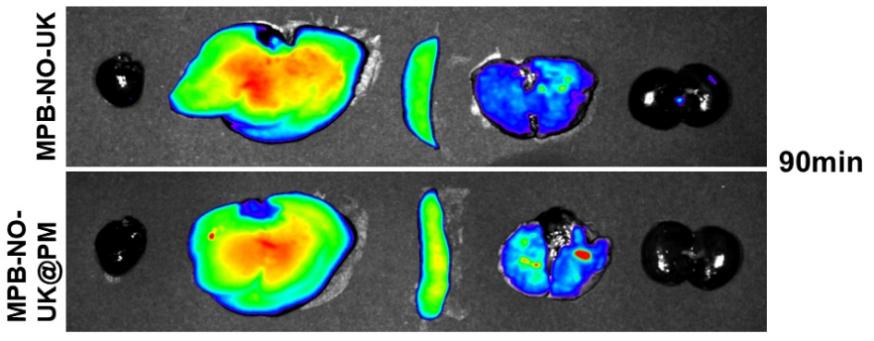


**Figure S13a.** Fluorescence imaging of the main organs after 90min.


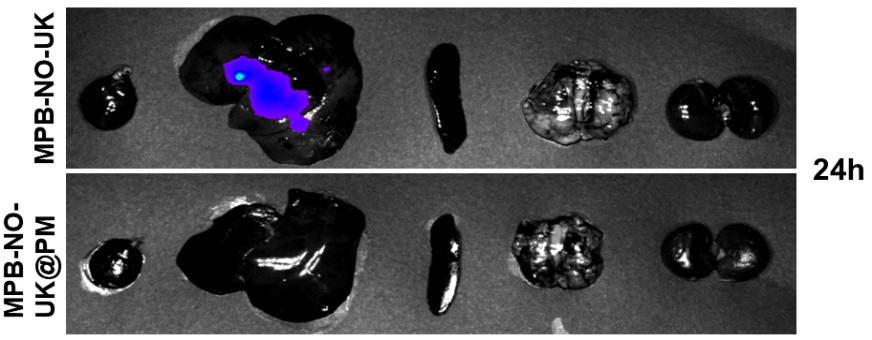


**Figure S13b.** Fluorescence imaging of the main organs after 24 h.


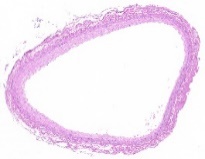

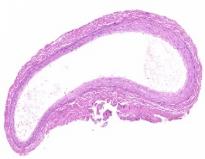

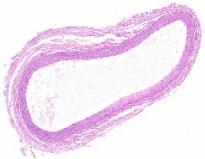


**Figure S14.** H&E staining of carotid thrombi 7 days after treatment of MPB-NO-UK@PM+NIR (*n* = 3).


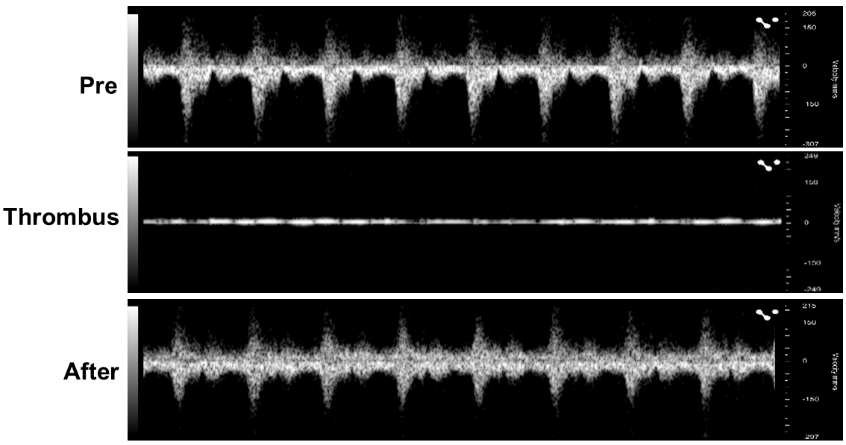


**Figure S15.** Doppler spectral signal images of rats before treatment, at the time of thrombus formation, and 7 days after treatment.
